# Exploring the multifaceted Dbf4-dependent kinase from temporal, spatial, and substrate repertoire perspectives

**DOI:** 10.1038/s42003-026-10512-5

**Published:** 2026-06-23

**Authors:** Lorenzo Galanti, Boris Pfander

**Affiliations:** 1 Cell Biology, TU Dortmund Life Science Center (DOLCE), Department of Chemistry and Chemical Biology, TU Dortmund University, Dortmund, Germany; 2 Present Address: DSB Repair Metabolism Laboratory, The Francis Crick Institute, London, UK

**Keywords:** DNA replication, Cell division, DNA recombination

## Abstract

The Dbf4‑dependent kinase (DDK), composed of the catalytic subunit Cdc7 and the regulatory subunit Dbf4, is a serine/threonine kinase traditionally defined as an essential activator of DNA replication. Here, we review DDK function from three complementary perspectives: its temporal regulation during the cell cycle, its spatial organization on chromosomes, and its expanding substrate repertoire. These perspectives reveal that DDK acts beyond DNA replication, targeting proteins involved in chromosome segregation, DNA damage responses and homologous recombination. Together, they redefine DDK as multifunctional genome integrity kinase which coordinates cell cycle progression and genome stability offering a unique therapeutic potential in cancer therapy.

## Abstract (web summary)

This review discusses how the Dbf4 dependent kinase (DDK) coordinates DNA replication, repair and chromosome segregation through temporal control, chromosomal localization and an expanding substrate repertoire

## Introduction

The Dbf4-dependent kinase Cdc7 (DDK) is an essential cell cycle kinase, conserved throughout eukaryotes. DDK is active as a heterodimer, consisting of the S/T kinase Cdc7 and the regulatory subunit Dbf4. DDK is most well-known as central regulator of DNA replication, but recent work suggests a more widespread function in genome maintenance. In this review, we discuss DDK function from three complementary perspectives - its temporal regulation, its spatial organization, and its substrate repertoire (Box 1). First, we consider the temporal regulation of DDK and discuss DDK as a cell cycle kinase. DDK is active from S to M phase of the cell cycle. Beyond its canonical role in replicative helicase activation and DNA replication origin firing, it phosphorylates substrates involved in mitosis and chromosome segregation, often in coordination with cyclin-dependent kinase (CDK). Second, we examine how subcellular and chromosomal localization, as well as docking to substrates and substrate complexes contributes to substrate selection and discuss DDK as a chromosomal kinase. Finally, we summarize current knowledge on DDK’s substrate repertoire and discuss the emerging view of DDK as a genome integrity kinase. Together, these perspectives highlight DDK as a multifunctional kinase whose temporal regulation, chromosomal localization, and substrate repertoire collectively coordinate cell cycle progression with the maintenance of genome integrity.

Box 1 Three perspectives of DDK function This review approaches DDK function from three distinct perspectives:

*Temporal perspective* - DDK activity is tightly linked to the cell cycle. It becomes active at the onset of S phase and remains active through G2 and mitosis before being downregulated by APC/C-dependent degradation of Dbf4. This timing allows DDK to control DNA replication, but also to activate genome integrity pathways that act post-replicatively.

*Spatial perspective* - DDK acts primarily as a chromosome-associated kinase. It localizes to replication origins, centromeres and replisomes through interactions with chromatin-bound factors. Local enrichment enables selective phosphorylation of proteins involved in DNA replication and chromosome organization, while phosphatases such as Rif1–PP1 counteract DDK activity.

*Substrate repertoire perspective* - Beyond the replicative helicase Mcm2–7, DDK phosphorylates proteins involved in replication stress responses, DNA repair and chromosome segregation. These substrates position DDK at the interface between DNA replication and genome stability pathways.

Together, these perspectives highlight DDK as a regulator of chromosome metabolism that coordinates DNA replication with genome maintenance across the cell cycle.

### The temporal perspective: DDK – a cell cycle kinase

**Fig. 1:**
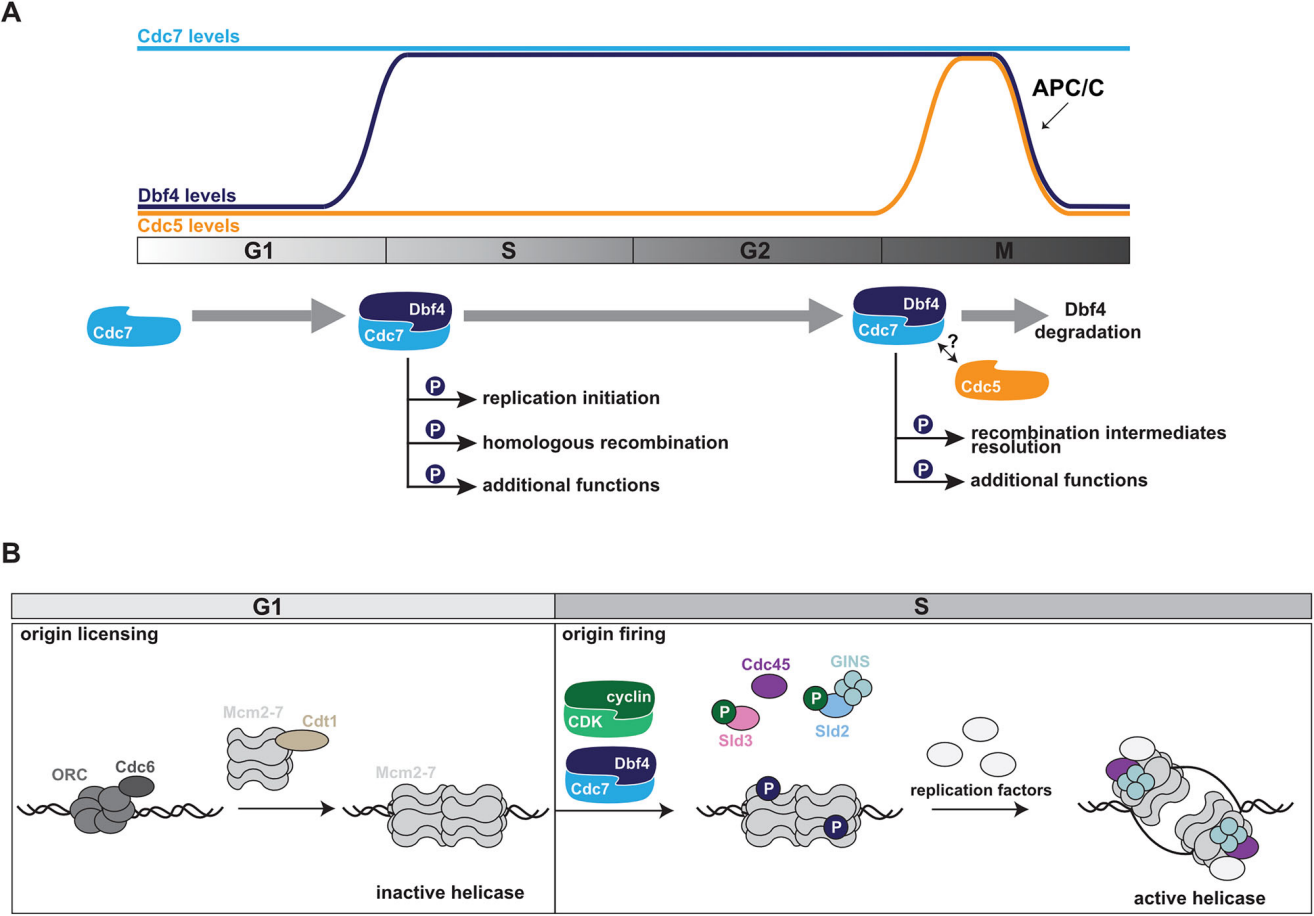
A cell cycle kinase – Temporal Regulation of DDK in *S. cerevisiae.* Temporal regulation is exemplified by the known regulation of budding yeast DDK. **A** Several factors control DDK activity during the cell cycle. Dbf4 is regulated by the ubiquitin-proteasome system with Dbf4-degradation being induced in M phase (anaphase) via APC/C-dependent ubiquitination. This results in active DDK from S phase to early M phase, which activates replication initiation, homologous recombination and additional functions. Additionally, in early M phase DDK interacts with Cdc5, which activates resolution of recombination intermediates and additional functions. **B** DDK participates in the cell cycle regulation of DNA replication initiation. Left - The first step – origin licensing – occurs in the G1 phase and in the absence of DDK activity and generates inactive precursors of the Mcm2-7 replicative helicase. Right – In S phase, these inactive helicase precursors are converted to active CMG helicases by origin firing. This second step is mediated by CDK and DDK activity.

DDK is highly regulated during the cell cycle and restricted to S, G2 and M phases (Fig. 1a). In budding and fission yeast, this regulation is achieved by APC/C-dependent degradation of Dbf4 from late-M to G1^1–6^. Similarly, in mammalian cells DBF4 levels are regulated during the cell cycle^7–11^. We can therefore consider DDK to be temporally regulated during the cell cycle and to mediate cell cycle phase-specific functions. Consistently, Cdc7 has first been identified as a cell cycle regulator by the Hartwell *cdc*-mutant screen in *Saccharomyces cerevisiae*^12^. Conditional *cdc7-*mutant yeast strains arrest their cell cycle after START (point of commitment for entering a new cell cycle), but with unreplicated DNA indicating that DDK is essential for S phase and DNA replication^12,13^.

Indeed, a key function of DDK is to activate DNA replication initiation during S phase and this function appears to be conserved from yeast to human^14–20^. Replication initiation is a two-step process, during which the replicative helicase is first loaded at origins of replication in the form of an inactive precursor (origin licensing, Fig. 1b). In S-phase, these helicase precursors become activated by phosphorylation and the association of accessory subunits (origin firing, (Fig. 1b)). Specifically, DDK functions during origin firing: it targets the catalytic Mcm2-7 core of the CMG (Cdc45-Mcm-GINS) helicase to alleviate auto-inhibition^19,21–23^ and generate binding sites for Sld3, a key origin firing factor^24^. Importantly, activating the replicative helicase constitutes the essential function of DDK, as specific mutations in the replicative helicase that bypass the need for DDK phosphorylation of Mcm2-7 (*mcm5-P83L (bob1-1), mcm4Δ74-174*) also bypass the essentiality of *CDC7* and *DBF4* genes and allow the corresponding mutants to proliferate^16,19^. These data have initially led to the view that DDK functions primarily in S phase driving DNA replication.

However, DDK is highly active until late-mitosis, when Dbf4 becomes degraded by the APC/C^1–7,9,10,17^, indicating that DDK might have additional functions after S phase. Indeed, budding yeast DDK has been shown to phosphorylate several proteins involved in genome stability^25–27^ and chromosome segregation in M phase^28^. It is currently unclear whether also human DDK has a widespread function in phosphorylating proteins in M phase, even if initial data suggest conservation^25^. A post-replicative function of DDK is not restricted to the mitotic cell cycle, as DDK has been shown to regulate meiotic chromosome segregation in budding yeast^29–35^. Therefore, DDK has clear functions after S phase in mitotic and meiotic cell cycles. Meiotic functions of DDK have been summarized elsewhere^36,37^, in this review we will instead focus on DDK’s function during mitotic cell cycles.

Notably, some of these late functions depend on DDK-binding to the mitotic cell cycle kinase Cdc5 in yeast^26,28,31,38,39^. It is plausible that DDK may recognize a distinct set of substrates when bound to Cdc5^26,28,31^. These data are therefore consistent with a model whereby DDK phosphorylates two pools of substrates: the first pool contains substrates such as the Mcm2-7 proteins, which are phosphorylated from S to M phase and depend specifically on DDK, while the second pool of substrates is phosphorylated specifically in M phase and depends on DDK, but potentially also Cdc5. Currently, it is unclear whether the physical interaction of DDK and Cdc5 is unique to budding yeast or evolutionarily conserved.

Another important functional interaction occurs with cyclin-dependent kinases CDK1/2 (referred to as CDK hereafter, Cdc28 in budding yeast). Through its cell cycle-regulated expression and APC/C-dependent degradation of Dbf4, DDK activity is temporally coupled to the activation/inactivation of CDK. Therefore, DDK and CDK are generally co-regulated during the cell cycle, even though CDK’s fine-grained regulation with several cell cycle transition-specific cyclins is missing for DDK^40,41^. While vertebrates express a *DBF4* paralog called *DRF1*, this factor appears to be of lesser importance for the control of DNA replication in somatic cells^42^, and at least in *Xenopus laevis* is developmentally controlled^43^.

Notably, CDK and DDK are not only active at the same time, but sometimes even phosphorylate the same substrates. One example is given by the regulation of homologous recombination in budding yeast (see “4.3 DDK and DNA double-strand break repair”), which was known for some time to be under CDK control^44–46^, but according to our recent data is under dual kinase control by CDK and DDK^25^. In some cases, CDK and DDK even target the same substrate interdependently and CDK catalyses a priming phosphorylation which is extended by DDK for a secondary phosphorylation step (examples include Dna2, Eco1, Mcm4/6, Mer2, Sld2 in budding yeast^25,27,33,34,47,48^). Further work will need to clarify whether DDK should be viewed as a cell cycle kinase that acts primarily in conjunction with CDK (dual kinase phosphorylation).

Additionally, several proteins are known in the budding yeast system which are simultaneously phosphorylated by DDK and Cdc5. This group of proteins includes Lrs4, Mms4, Rec8 and Sld2^26,27,29,31^, but priming phosphorylation or interdependency has not been observed for any of these substrates. These data therefore suggest that these phosphorylation events have independent functions, even though the kinases interact.

Overall, the cell cycle kinase model has been most influential and invaluable for our understanding of DDK biology. However, there appear to be nuanced differences in the cell cycle function of DDK, which need further exploration. For example, DDK is essential for S phase and DNA replication in budding yeast^16,19^ as well as in Xenopus replication systems^49^, but a recent study suggested that in humans and mice DDK is not strictly required for proliferation^50^.

### The spatial definition: DDK – a chromosomal kinase

**Fig. 2:**
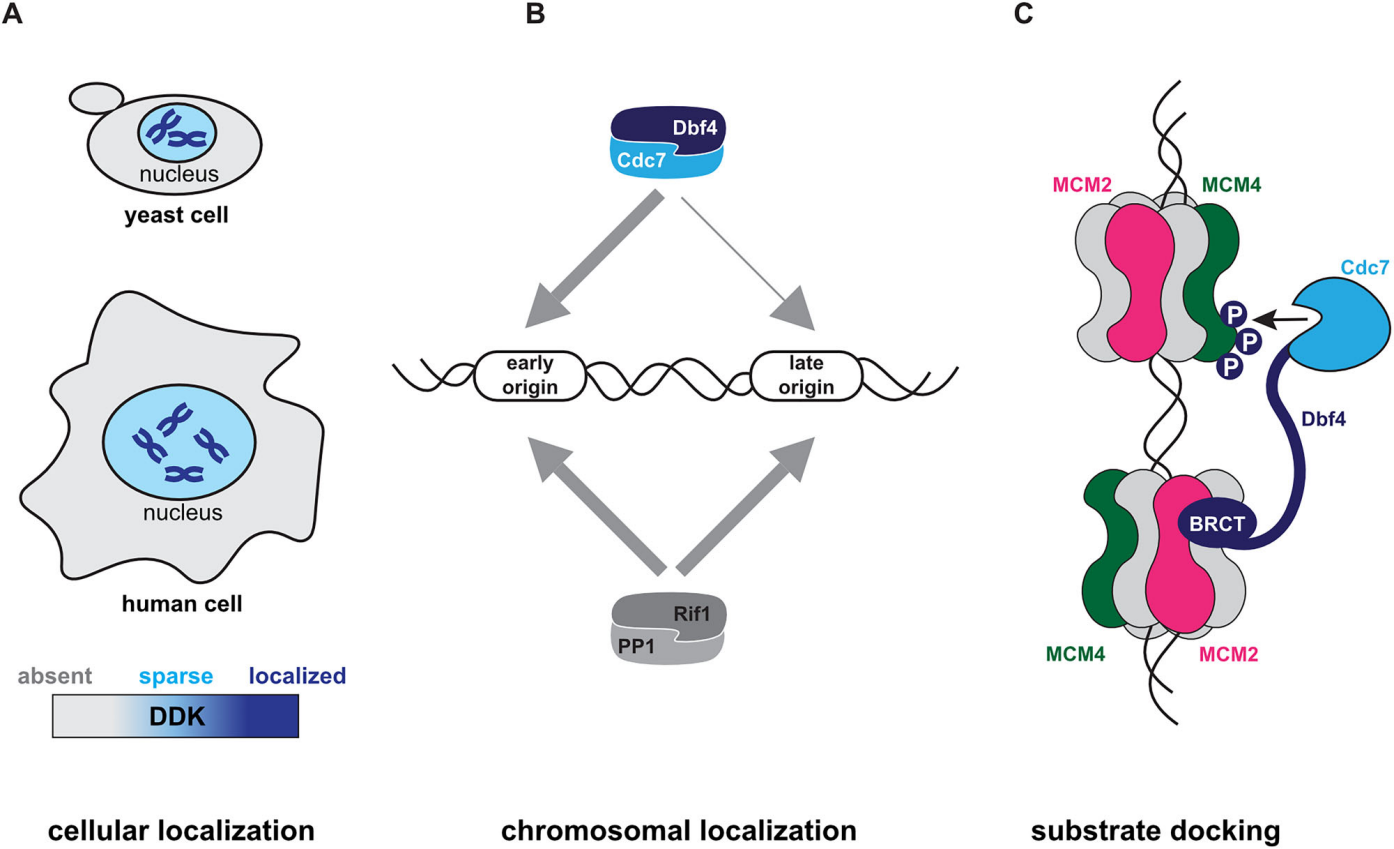
A chromosomal kinase – Spatial Regulation of DDK. **A** DDK is primarily localized to the nucleus in budding yeast and mammalian cells, where it is sparsely localized to the nucleoplasm and primarily found localized to chromosomes. **B** DDK shows sub-chromosomal specificity and localizes with preference to early replicating origins, but not to late origins. In contrast, the DDK-antagonizing Rif1-PP1(Glc7) appears to be equally localized to replication origins. Collectively, both mechanisms generate a gradient of DDK-activity important for the temporal programme of replication initiation. **C** DDK utilizes a substrate docking mechanism to phosphorylate inactive Mcm2-7 helicase precursors in budding yeast. Dbf4 binds, among other sites to Mcm2 with its BRCT domain. This binding positions the complex to phosphorylate Mcm4 across the Mcm2-7 double hexamer interface. Cartoon conceptually adapted from Greiwe et al.^22^. While DDK targets Mcm2-7 double hexamers, separated hexameric MCMs are shown to visualize the trans-activity of DDK more clearly.

A second key attribute of DDK is its sub-cellular localization. In yeast and human cells, DDK localizes predominantly to the nucleus (Fig. 2a)^7,17,18,51–54^. Additionally, using biochemical fractionation, DDK was found to specifically localize to chromatin in yeast cells and Xenopus egg extracts^55–60^. Moreover, DDK chromatin binding even correlates with its DNA replication initiation function as DDK was shown to preferentially localize to early-firing origins in yeast and localization to origins coincided with the timing of their activation^58,61,62^. Interaction with Mcm2-7 proteins is likely contributing to recruitment to replication origins^20,57,59,63,64^. Furthermore, an interaction with the fork progression complex observed in both budding and fission yeast suggests that DDK is also recruited to replisomes (see “4.2. DDK and replication stress”)^32,65,66^.

Accordingly, these data support a model whereby specific regions form dynamically along chromosomes, which are marked by increased DDK activity. Subcellular localization may therefore be involved in DDK substrate selection (Fig. 2B). Such regions are likely to be shaped not only by phosphorylation, but also by phosphorylation reversal, which in the case of DDK substrates, is typically catalysed by the PP1 phosphatase in complex with Rif1 that acts to reverse phosphorylation of Mcm2-7 proteins across eukaryotes^49,67–71^. Importantly, Rif1 itself is highly localized along chromatin, including replication origins, telomeres and DNA damage sites and – at least in human cells – developmentally regulated genes^72–79^. As such, we expect that the Rif1-PP1-dependent antagonism of DDK phosphorylation is crucial for shaping DDK regulation along chromosomes (see “4.4 Regulation of DDK substrates by Rif1-PP1”).

DDK’s role as a chromosomal kinase is underscored by its phosphorylation of key chromosome architectural proteins. Substrates include histone chaperones and chromatin remodellers, such as human CAF1 and budding yeast INO80-C, both of which undergo DDK-dependent regulation during DNA replication^55,80^. Furthermore, DDK was found to regulate the cohesin complex in different organisms, even though it appears to be acting on distinct proteins. In budding yeast, DDK phosphorylates the cohesion establishment factor Eco1, creating a cell-cycle-dependent degron, which mediates S phase specificity of Eco1 function^47,81^. DDK also phosphorylates Rec8, the α-kleisin subunit of the meiosis-specific cohesin variant, and promotes its cleavage by separase^29^. In the *Xenopus* egg extract system and also human cells, DDK was shown to act on the cohesin loader SCC2-SCC4 and promote cohesin loading, suggesting a role in establishing chromosome architecture^82,83^. Lastly, the cohesion regulator PDSB5B is phosphorylated by DDK as well, but this mechanism still awaits detailed functional characterization^84^.

DDK localization to centromeres furthermore highlights its role as a chromosomal kinase^58^. Accordingly, budding yeast DDK phosphorylates the centromeric H3 variant Cse4 (ortholog of CENP-A), as well as the kinetochore proteins Mif2 (directly binds the centromeric nucleosome) and Ctf19 (part of the COMA complex), whereby Cse4 phosphorylation is involved in centromere determination in M phase and Ctf19 phosphorylation is critical for establishment of centromeric cohesion in S phase^28,85–87^. Also in human cells, DDK acts at centromeres, where it leads to phosphorylation of TOP2A and mediates release of TOP2A from centromeres^54^.

How DDK localization translates into the phosphorylation of specific substrates is a matter of ongoing research, as is the substrate recognition mechanism itself. Biochemical experiments, as well as detailed studies of DDK-dependent phosphorylation sites on Mcm2-7 and other proteins suggest that DDK preferentially phosphorylates S/T motifs that harbour a negatively charged residue at the +1 position (D, E, phospho-S)^25,27,47,48,88^. However, phosphoproteomic analysis of DDK-dependent phosphorylation sites in yeast did not strongly enrich this consensus motif for the bulk of potential substrates^25,84^, suggesting substrate recognition may likely involve other factors. Indeed, biochemical studies on the substrate-recognition mechanism have shown that DDK employs a substrate docking mechanism (binding the substrate at a site which is not the phosphorylation site) to phosphorylate the Mcm2-7 helicase precursor (Fig. 2c)^20,63,64,89^. Specifically, docking involves the N-terminal BRCT domain of Dbf4, but also other parts of DDK and has been shown in molecular detail by structural studies^21–23^.

Like many chromosomal proteins, DDK is SUMOylated, whereby monoSUMO modification allows DDK activity, while SUMO chain modification leads to ubiquitination and proteasomal degradation^90,91^. Notably, SUMOylation typically mediates protein-protein interactions and may therefore play a role in substrate recognition by DDK and docking.

Future studies need to address whether substrate recognition by a docking mechanism is a prevalent feature also for the other substrates. If it is, we suspect that such substrate-kinase complexes will be formed specifically on chromosomes where there is a high local concentration of substrate and kinase. Spatial regulation of DDK is therefore likely to be a crucial feature of DDK function, and DDK may be viewed as a chromosome-targeted kinase.

### The substrate-based definition: DDK – a genome integrity kinase

Next to its temporal and spatial regulation, we can also approach DDK function by focusing on its phosphorylation substrates. Many DDK targets have established roles in genome integrity. We will therefore focus on the perspective of DDK as a genome integrity kinase. The majority of validated DDK substrates in budding yeast have established functions in genome integrity, including pathways such as DNA replication, DNA damage response, DNA recombination and chromosome segregation and meiotic DSB repair^16,20,25–29,31–34,59,86,90,92,93^. Additionally, recent unbiased analysis of the DDK phosphoproteome in yeast suggested further DDK substrates in DNA repair, DNA recombination, DNA damage response and chromatin remodelling pathways^25,55^.

Given its cell cycle regulation and chromatin association, DDK is well-positioned to regulate genome integrity pathways. Consistently, DDK regulates the response to DNA damage and replication stress. For example, budding yeast DDK interacts with the checkpoint kinase Rad53 and it was shown early on that the presence of DNA damage and replication stress leads to a reduction in Mcm2-7 phosphorylation^59,89,94–97^. This has led to a model whereby DDK might be generally inhibited after DNA damage and replication stress^95,97^. Alternatively, we hypothesize that upon DNA damage and replication stress, DDK may change its substrate spectrum away from Mcm2-7 (and other factors involved during undisturbed cell cycles) and towards proteins involved in DNA recombination, repair and the response to replication stress. Interaction with and phosphorylation by checkpoint kinases may be involved in such a putative substrate shift.

As such, we view DDK as a regulator of genome integrity that acts during undisturbed cell cycles and in response to DNA damage and replication stress. In the next chapters of this review, we will therefore focus on different aspects of DDK’s function as a genome integrity kinase.

### DDK and DNA replication

DDK is most well-known for its function in coupling replication initiation, more specifically origin firing, to S phase. This function has been discussed extensively in several excellent reviews on replication initiation and has revealed mechanistic features that may or may not serve as paradigms for DDK function^40,98^. In budding yeast, DDK phosphorylates specifically loaded Mcm2-7 double hexamers, the inactive precursor of the replicative helicase^21–23,63^. Interestingly, the complex is phosphorylated at multiple sites, but phosphorylation is directed with remarkable specificity to the N-terminal tails of Mcm4 and Mcm6^21–23,48,59,63,92,99^. Phosphorylation of these sites promotes helicase activation via a dual mechanism, by (i) relieving an autoinhibition mediated by the Mcm N-terminal tails^19^ and by (ii) promoting a docking site for the helicase assembly factor Sld3, which promotes an interaction with Cdc45 and (indirectly) GINS^24^. Sld3 appears to be phosphorylated in a DDK-dependent manner as well^100^, but the molecular mechanism remains to be determined. Notably, phosphorylation of the Mcm2-7 complex by DDK appears conserved throughout eukaryotic evolution^5,7,17,18,101–105^. Moreover, DDK targets *Xenopus* Treslin-MTBP (the orthologues of Sld3 and Sld7 in budding yeast), indicating further evolutionary conservation in DDK control of DNA replication^106,107^.

DNA replication follows a temporal programme, with individual replication origins becoming activated with specific timing during S phase. Interestingly, DDK may be involved in generating this temporal programme. In budding yeast, DDK was found to be a limiting factor for DNA replication, and was proposed to associate first with early replicating origins and subsequently be recycled to late replicating origins^108^. A similar phenomenon was also observed in human cells, where *CDC7*-analogue sensitive cell lines revealed that late replicating regions required higher DDK activity compared to early replicating regions^84^.

Generally, the role in replication initiation is a key example of functional cooperation of DDK and CDK (Cdc28-Clb5/6, Cyclin A-CDK2 in human cells), which collectively promote helicase activation and thereby origin firing^40,98^. This two-kinase mechanism of replication activation appears to be evolutionary conserved throughout eukaryotes^109^. Of note, while both DDK and CDK are essential for DNA replication in yeast and *Xenopus* replication systems^16,19,49^, a recent study suggests that DDK is not strictly required for proliferation in non-transformed human and mouse cells^50^. The authors used different systems (analogue-sensitive kinase alleles, auxin-inducible degrons as well as knock-outs) to interfere with DDK activity/stability and observed proliferation in different non-cancerous human cell lines and mice tissues despite a strong decrease or even absence of DDK activity, suggesting that DDK can be dispensable for proliferation. Notably, the same study found Cyclin-B CDK1, known to be mainly active in late S-phase/mitosis to act redundantly with DDK for the initiation of DNA replication, and lack of both CDK1 and DDK was found to be lethal^50^. This finding suggests functional redundancy of the two cell cycle kinases and that – despite not strictly essential – DDK is an important driver of proliferation. Notably, DDK was found to be required for DNA replication and cell proliferation in different cancerous mammalian cells and tumours^103,110–112^. This suggests that cancer cells can become addicted to DDK. It is possible that the phenotypic differences observed in cancerous or non-cancerous cells may rely on the replication initiation function of DDK, additional functions in response to replication stress (see “4.2 DDK and replication stress”) or a combination of both.

Curiously, in budding yeast, DDK phosphorylates yet another replication initiation factor, Sld2, to trigger its degradation in M-phase^27^. Collectively, phosphorylation of Sld2 by several mitotic kinases creates a phosphodegron, which leads to Dma1/2-dependent ubiquitination and proteasomal degradation, a mechanism that is involved in inactivating Sld2 before the next cell cycle and onset of origin licensing, respectively^27^. How the specific timing of this degradation is achieved is a matter of ongoing investigation.

DDK appears to also have a more wide-spread role in chromatin replication. A recent paper shows DDK phosphorylation of the INO80-C nucleosome remodelling complex in budding yeast^55^, which appears important for INO80-C nucleosome spacing activity and for chromatin replication in vitro, as well as for S phase progression in vivo. Moreover, earlier work showed phosphorylation of CAF1 in human cells, suggesting a role in replication-coupled chromatin assembly^80^.

### DDK and DNA replication stress

**Fig. 3:**
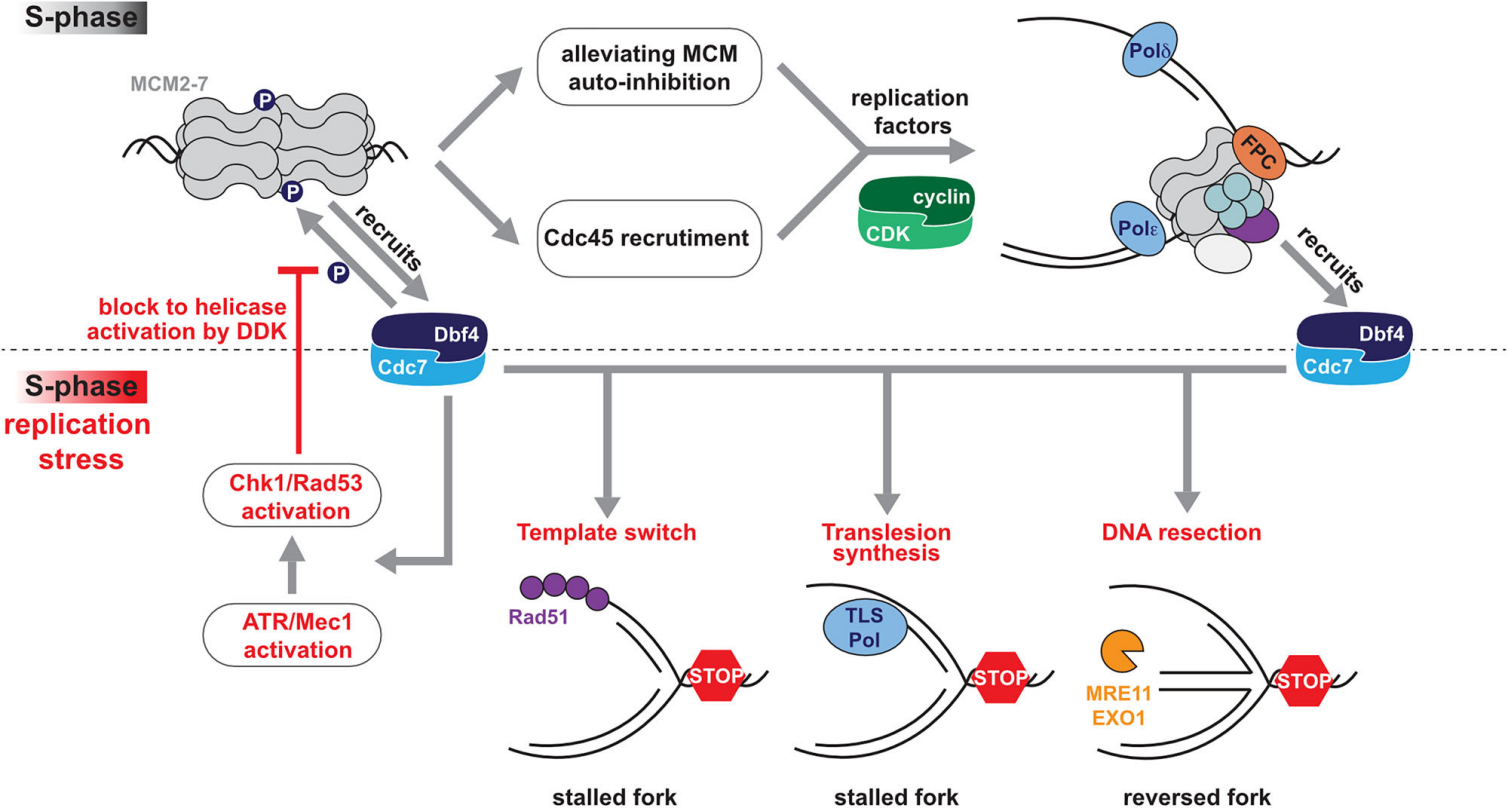
DDK as a hub of replication control. Top – In every S phase DDK phosphorylates Mcm2-7 helicase precursors. These phosphorylation events alleviate the autoinhibition of the helicase and lead to the recruitment of the accessory helicase subunit Cdc45. Additional phosphorylation events by CDK lead to activation of the replicative helicase and origin firing. On chromosomes, DDK is recruited to Mcm2-7 helicase precursors (left), but also to active replisomes (right). Bottom – DDK is involved in several replication stress response mechanisms. Upon induction of replication stress, apical checkpoint kinases (ATR in human/ Mec1 in yeast) and effector checkpoint kinases (CHK1 in human/ Rad53 in yeast) are activated and phosphorylate DDK, which leads to inhibition of helicase activation at origins. Additionally, DDK is actively involved in promoting signalling of the DNA damage checkpoint, as well as DNA replication restart, translesion synthesis (TLS) and DNA resection of fork structures.

Many factors impede the progression of replisomes, leading to DNA replication stress. DDK is critically required for the cellular response to replication stress and we can subdivide at least three different functions, in which DDK is actively or passively involved (Fig. 3). First, DDK is targeted by checkpoint kinases to inhibit further origin firing; second, DDK is a regulator of replication fork stalling; third, DDK facilitates the DNA damage checkpoint response.

(i) A checkpoint target – DDK is targeted by checkpoint kinases in both yeast and human cells and this prevents further replication initiation events in conditions of replication stress. DNA damage checkpoint kinases in budding and fission yeast phosphorylate Dbf4, which leads to a reduction in DDK-dependent phosphorylation of Mcm4 and Mcm6^59,95,97,99,101,113^. Initial interpretations suggested that DDK kinase activity was inhibited in general, but cryo-EM studies showed that Rad53-mediated phosphorylation of DDK may specifically affect DDK binding to Mcm2-7 helicase precursors^21,22^. Additionally, Rad53-binding may also sterically antagonize DDK binding to Mcm2-7^89^. Also in metazoans, DDK is targeted by checkpoint kinases to prevent further DNA replication in conditions of replication stress. Induction of the ATR/ATM-CHK1-mediated checkpoint cascade was shown to target DDK in *Xenopus* egg extracts and human cells^60,114–116^. Also in metazoans, the inhibition of DDK was suggested to block further replication initiation, but additionally, the activity of DDK appeared to be required to preserve replication fork stability, suggesting that DDK is not entirely inhibited, but may rather be directed to other substrates^60,114–119^. Therefore, a model emerges whereby specifically inhibition of DDK-dependent activation of the replicative helicase is critical for the replication stress response across eukaryotes. While DDK activity may be dampened upon phosphorylation, it is clearly still required to phosphorylate additional proteins to elicit the replication stress response.

(ii) A regulator of DNA replication stress – DDK association with replisomes suggests it can phosphorylate proteins at (stalled) replication forks^32,65,66,120^. Consistently, active functions of DDK in response to replication stress have been identified, for example, in translesion synthesis (TLS) and error-free postreplicative repair (Fig. 3). Translesion synthesis involves specific translesion DNA polymerases (TLS polymerases) to overcome replication blocking DNA lesions^121^. Upon induction of replication stress in human cells by agents such as hydroxyurea (HU), UV-C light and mitomycin C, DDK appears to facilitate the recruitment of the TLS polymerase Pol η at stalled forks by a mechanism that relies on DDK-phosphorylation of the ubiquitin ligase RAD18^10,122^. Also in budding yeast, DDK may be involved in activating TLS as DDK-deficient yeast strains show low mutation rates – a hallmark of TLS^93^. Curiously, in this context, DDK appears to promote the function of a different TLS polymerase, Pol ζ^93^. Therefore, while DDK appears to regulate TLS in different eukaryotes, the exact mechanisms appear to differ.

Moreover, a function for DDK in error-free postreplicative repair was identified in budding and fission yeast as well^90,123^ and possible substrates could be narrowed down to Rad51 in the case of budding yeast DDK^90^. In particular, it was shown that mono SUMOylation of DDK promotes phosphorylation of Rad51 and the stabilization of Rad51 on ssDNA at stalled replication forks, therefore promoting a template switch^90^.

**Fig. 4:**
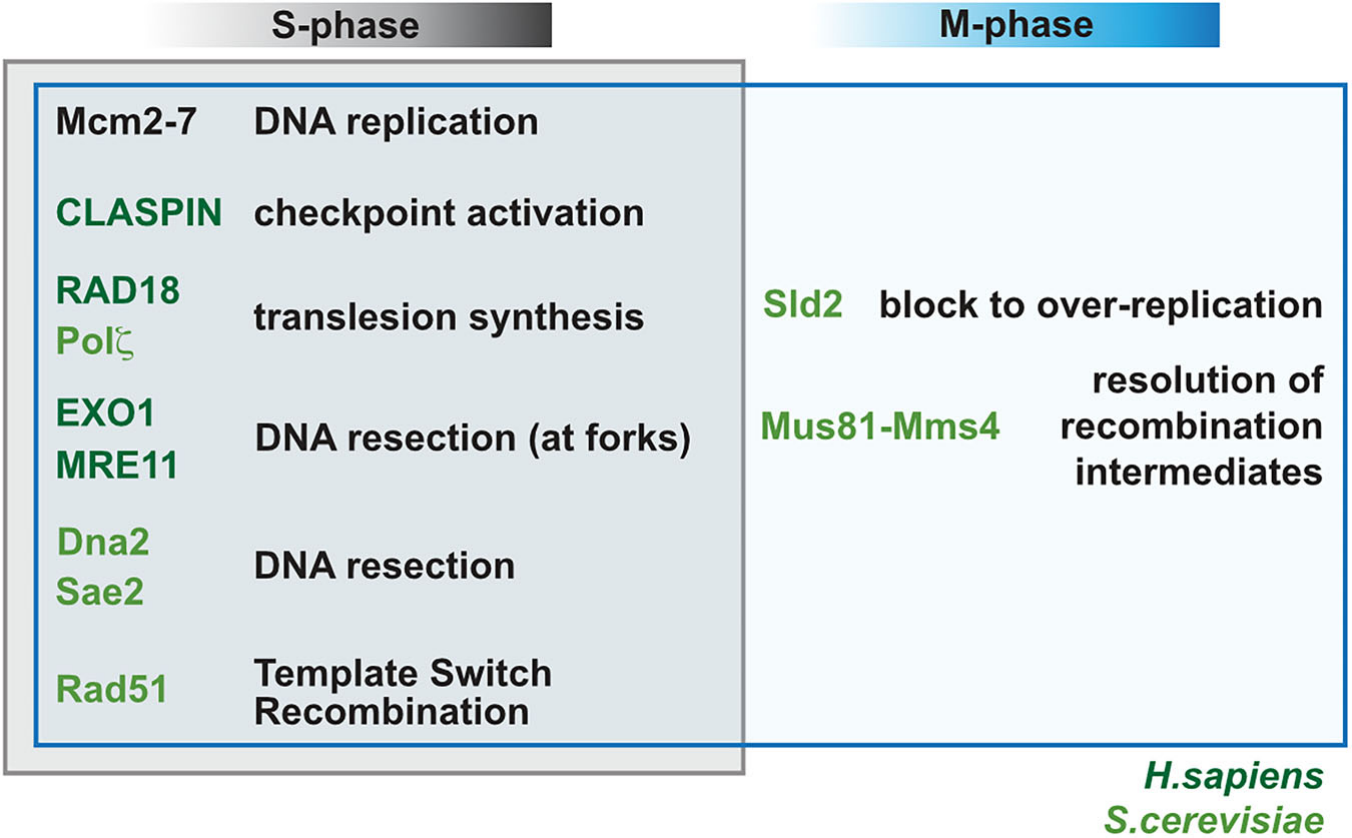
A genome integrity kinase - DDK targets key genome integrity factors. Most DDK substrates characterized in mitotically dividing cells function in different genome integrity mechanisms. Most substrates are already phosphorylated in S phase and maintain phosphorylation in M-phase, while unique M-phase-specific phosphorylation substrates exist, at least in budding yeast. DDK substrates in human are shown in dark green and DDK substrates in yeast are shown in light green, evolutionary conserved DDK substrates are shown in black.

An alternative response to replication stress involves fork remodelling to reverse forks. As reversed forks expose a double-stranded DNA end, they may become subject to DNA end resection. In human cells, DDK appears to regulate resection of reversed forks, in particular when fork protection mechanisms are impaired, for example in *BRCA*-deficient cells or upon checkpoint inhibition^120,124^. In this context, chemical inhibition of DDK reduces the accumulation of ssDNA at reversed forks, suggesting that DDK is an activator of resection (^120,124^, see “4.3 DDK and DNA double-strand break repair” and Fig. 4). A complication of all studies of replication stress is the intricate entanglement of DNA replication, replication stress and checkpoint mechanisms. In the context of fork reversal and BRCA-deficiencies, DDK promotes checkpoint activation, but it is currently unclear whether this is a consequence of resection activation or may reflect a more direct involvement in checkpoint signalling (^84,120,124,125^, see “(iii) A regulator of checkpoint signalling”). A direct role in resection was suggested by biochemical experiments, where human DDK was found to directly phosphorylate human EXO1^124^, a long-range resection factor that was already known to be regulated by CDK^126^. Consistent with a role in resection of reversed forks, it was also shown that chemical inhibition of DDK, or use of *CDC7* analogue sensitive (*CDC7-as*) mutants led to a reduction of resection at reversed forks, in particular in BRCA mutant cell lines^120^. In this study, DDK was shown to act on the MRE11 nuclease, given an epistatic relationship between CDC7 inhibition and MRE11 silencing and the loss of an HU-dependent MRE11 phospho-shift on gels upon DDK inhibition^120^. Furthermore, a second study using *CDC7*-analogue sensitive, *BRCA*-proficient cell lines suggested a role of DDK in stabilizing and restarting stalled replication fork structures^84^. In this study, no defect in fork resection was found upon DDK inhibition, a result that can be explained by the differential BRCA status in the investigated cell lines^84,120^. Finally, additional DNA repair/replication proteins were found to be targeted by DDK using phosphoproteomics experiments, including MERIT40 (a subunit of the BRCA1-A deubiquitinase complex) and PDS5B (an accessory subunit of cohesin)^84^. DDK-dependent regulation of DNA end resection factors is also seen in budding yeast^25^, but so far a role in the response to replication stress has not been investigated in this system (see “4.3 DDK and double-strand break repair”).

Therefore, DDK has been found to promote different DNA damage tolerance mechanisms and it generally may be involved in pathway choice. Along these lines, a recent budding yeast study suggested that DDK favours ssDNA gap filling and replication fork restart via mechanisms such as TLS^127^.

(iii) A regulator of checkpoint signalling – the interpretation of DDK phenotypes in the cellular responses to replication stress is complicated by the manyfold functions of DDK in DNA replication and DNA replication stress. For example, DDK-deficient cells show reduced checkpoint activation in various eukaryotic systems^19,113,128–130^, but these phenotypes could be due to direct effects (DDK being involved in checkpoint signalling), or indirect effects such as defective replication initiation generating fewer replication forks that in turn result in less checkpoint activation^131,132^ or a mixture of both. Conversely, it cannot be ruled out that replication stress phenotypes in DDK mutant cells result at least partially from checkpoint defects. In budding yeast, DDK was proposed to be directly involved in checkpoint activation, due to impaired checkpoint signalling in mutant backgrounds (*mcm5-P83L (bob1-1)* and *mcm4Δ74-174*) that bypass DDK’s function in DNA replication initiation^19,129^. In human cancer cell lines siRNA depletion of CDC7 or chemical inhibition of DDK were also shown to reduce or delay checkpoint activation^125,128^. A direct involvement of DDK in phosphorylating the checkpoint mediator CLASPIN was proposed, leading to a phosphorylation-dependent interaction between CLASPIN and CHK1, which in turn promotes checkpoint activation^125,128,133^. Notably, another acidic kinase (CK1) was proposed to have similar functions in the phosphorylation of CLASPIN, suggesting functional overlap of DDK and CK1^133,134^. Another example of direct involvement in checkpoint signalling comes from fission yeast, where upon replication stress DDK phosphorylates the Rad9 subunit of the 9-1-1 checkpoint clamp^135^. Interestingly, this phosphorylation appears to inhibit 9-1-1 interaction with RPA, suggesting DDK acts as a negative regulator of checkpoint signalling^135^. Overall, these data suggest that DDK has a widespread role in regulating the replication stress response in addition to its essential function during replication initiation.

### DDK and DNA double-strand break repair

Eukaryotes have evolved a number of DSB repair mechanisms that range from end-joining-based mechanisms, such as non-homologous end-joining (NHEJ) and microhomology-mediated end-joining (MMEJ) to template-dependent homologous recombination (HR)^136^. Importantly, the choice of the DSB repair mechanism is largely guided by the initial processing step – DNA end resection, which involves several nucleases that are also involved in the processing/resection of replication fork structures. Consistent with the view that DDK acts as a genome integrity kinase, there is increasing evidence for a direct role of DDK in the repair of DNA damage, specifically DSBs. First, DDK was shown to be important in regulating the repair of DSBs via break-induced replication (BIR), an HR-dependent repair mechanism which can be used to repair one-ended DSBs^137^. Interestingly, this function was independent of DDK’s function in replication initiation and phosphorylating the Mcm2-7 complex, as BIR phenotypes could not be fully rescued by the *mcm4Δ74-174* bypass mutant^138^, consistent with BIR being driven by repair synthesis, which is mechanistically distinct from canonical DNA replication. More recently, DDK was found to be important for DSB repair by homologous recombination (HR), in both yeast and human cells^25,139,140^. Chemical inhibition of DDK was shown to lead to defective HR-mediated repair in human cells, consistent with an observed additive effect on cell viability when DDK-inhibited cells were treated with DNA-damaging agents^139,140^. Moreover, yeast cells depleted of DDK are defective in DSB repair by HR, and DDK appears to be important to promote DNA end resection initiation and elongation, via phosphorylation of the nuclease complexes Sae2-MRX and STR-Dna2, respectively^25^. Notably, it is possible to synthetically activate DDK in G1 cells, where DDK is normally inactive and HR is downregulated. Upon DDK induction in G1 cells, Sae2 was shown to become phosphorylated, indicating the synthetic activation of DDK. Under these conditions, an increase of both resection and HR could be measured to modest extent^25^. A specific role of DDK in promoting DNA end resection and HR was also observed in human cells^25,140^. DDK therefore, appears important to process the broken ends at DSBs and promote repair via HR, with functions conserved throughout evolution.

DDK’s functions in regulating HR are, however, not restricted to the initial steps of HR. In budding yeast, DDK also phosphorylates Mus81-Mms4^26^, which in turn is important for the resolution of Holliday Junctions in the final steps of HR^141^. The specific roles of DDK in regulating HR are therefore still under investigation and so far only a limited number of target proteins have been investigated; even though phosphoproteomic analysis has identified a more widespread involvement of DDK in phosphorylating DNA repair proteins in both yeast and human cells^25,55,84^.

### Regulation of DDK substrates by Rif1-PP1

The aforementioned functions of DDK are typically counteracted by dephosphorylation mediated by the Rif1–PP1 phosphatase complex. PP1 phosphatase is known to act on DDK substrates and is thought to be a major antagonist of DDK phosphorylation. In budding yeast, DDK phosphorylation is counteracted specifically by Rif1-Glc7 and in human cells by RIF1-PP1. In yeast cells, the Rif1-Glc7 interaction is important to promote a Glc7-dependent dephosphorylation of MCM, therefore counteracting DDK phosphorylation of MCMs and avoiding premature/de-regulated origin firing^68,71,100,142^. Interestingly, the interaction between Rif1 and Glc7 (required for the dephosphorylation) was suggested to be downregulated upon DDK-mediated phosphorylation of Rif1, suggesting mutual inhibition^68,71,100^. Similar interactions and PP1-mediated dephosphorylation of MCM proteins were also identified in *Xenopus* egg extracts and human cells and were shown to involve RIF1-dependent PP1 recruitment^49,67,70^. Reinforcing the view of DDK-PP1 antagonism, removal of Rif1/RIF1 was found to partially compensate for defects caused by loss or inhibition of DDK, in both yeast and human cells^67,68,71,100^. Loss of RIF1 was also shown to impair the ability of cells to prevent further firing of replication origins in conditions of replication stress^67^.

More recently, the RIF1-PP1 complex was shown to regulate replication fork stalling as well. In human cells, loss of RIF1 led to enhanced DNA2-dependent resection of reversed replication forks^143,144^. Broadly, these findings are consistent with a DDK-dependent upregulation of resection at reversed forks, even though so far there is no evidence for DDK-dependent regulation of DNA2 in human cells. RIF1 per se, even without PP1, is known to counteract resection at DSBs or stalled replication forks^72–74,79^. In contrast, the protective DNA2-antagonising function of Rif1 was shown to be dependent on its interaction with PP1^143,144^, therefore being more similar to the Rif1-PP1-dependent dephosphorylation mechanism found for MCM2-7 proteins. Currently, the exact phosphorylation/dephosphorylation mechanism at forks remains unclear, but could rely on dephosphorylation of DNA2 directly, or of its accessory helicase WRN^143,144^. The kinase(s) involved in the activation of DNA2-mediated resection at replication forks in human cells are currently unknown, but could involve CDK or DDK given that budding yeast Dna2 is phosphorylated by both enzymes^25,44^. In contrast, DDK inhibition did not have an effect on nascent DNA resection as measured via DNA fibres from RPE1 cells^84^ highlighting that the role of DDK in regulating resection at reversed forks can vary depending on the cell background and conditions^84,120,124^.

### DDK in the clinic

Given the different roles of DDK in the maintenance of genome stability, DDK has become an interesting candidate to be targeted in cancer therapy. Targeting DDK is a promising strategy for different reasons. First, DDK is overexpressed in different types of cancers (e.g., glioblastoma, non-small cell lung cancer, Wilms Tumours, hepatocellular carcinoma, squamous cell carcinoma), where DDK overexpression is often correlated with p53 or Rb inactivation and could be a response to increased replication stress^145–147^. It is therefore considered a novel biomarker and moreover, may offer a promising therapeutic strategy, whereby DDK inhibition may reduce cellular proliferation and tumour growth^146^. Second, DDK was required for the proliferation of several tested cancer cell lines, but is seemingly not strictly required for the proliferation of non-cancerous cells and tissues^50,103,110,112,148^. This may suggest that DDK is essential specifically for the proliferation of cancer cells and as such DDK inhibition may allow selective killing. Third, as highlighted in this review, recent research has established DDK as a genome integrity kinase. Targeting DDK may therefore be particularly promising as it will hit both proliferation and genome integrity pathways. Its functions in genome integrity may also be crucial for combination therapies with chemotherapeutics or drugs targeting genome integrity pathways. Overall, DDK is therefore an attractive target for clinical inhibition, providing promising mono and combination therapies.

Different inhibitors of DDK have been developed, and some have been/are being tested in clinical trials^146,149^, including the recently developed compound TAK-931^103,139^. TAK-931 was shown to have additive effects on cell proliferation and survival when coupled to the induction of DNA damage^139^. Interestingly, and consistently with the proposed role of DDK in regulating HR-mediated repair, inhibition of DDK was shown to additively reduce tumour growth in xenograft models of breast and ovarian cancer when combined with PARP inhibitor treatment^139^. Given that some of the analysed xenograft models had wild-type genes of *BRCA1* and *BRCA2*, the synthetic lethality of combined DDK- and PARP-inhibition suggests that inhibition of DDK induces an HR-deficiency phenotype (BRCAness^139^,). Moreover, DDK inhibition by XL413 or TAK-931 induces a senescence phenotype that triggered inflammatory responses in cell-culture-based cancer cell models, as well as a myeloma mouse model^150–152^. Consistently, DDK inhibition was found to act synergistically with immune checkpoint inhibitors (anti-mPD-1 and anti-CTLA-4 specifically^151^). Moreover, XL413 showed synergism with anti-PD-1, as well as mTOR inhibitors in mouse xenograft models for hepatocellular carcinoma^152,153^. DDK inhibition therefore, appears to be also promising in combination with mTOR inhibition or immune checkpoint therapy.

Currently, the aforementioned addiction of cancer cells to the presence of active DDK is still a poorly understood phenomenon, yet highly promising for cancer therapy^153^. Consistently, TAK-931 leads to a reduction in cell proliferation and tumour growth in patient-derived xenograft models and the antiproliferative effect of DDK inhibition appears to be stronger in cancer cells, particularly p53-negative, compared to non-transformed cells, as non-transformed ones show only a transient and reversible arrest of the cell cycle upon depletion of CDC7^50,103,110–112^. Furthermore, with regard to possible side effects, an overall inhibition of cell proliferation upon DDK inhibition would be a major concern. As such, the recently proposed dispensability of DDK for DNA replication and proliferation in non-cancerous cells and tissues would suggest that overall toxicity and thereby non-specific side-effects could be minimal^50^. These findings suggest a therapeutic window could be found for DDK inhibitors, where overall toxicity and side effects are minimal. In the specific case of TAK-931, a phase I and II clinical trial suggested a good safety profile^154^, but the corresponding programme has been discontinued at Takeda Pharmaceuticals. Furthermore, a phase I clinical trial of an unrelated compound called SGR2921 (Schrödinger) has been discontinued following Serious Adverse Events. Therefore, alternative drug designs could become important for therapeutic DDK-inhibition. The known DDK inhibitors all target the catalytic centre of CDC7, making off-target effects on other kinases likely. For example, two commonly used DDK inhibitors, PHA-767491^110^ and XL-413^155^, were shown to elicit different cellular responses^156^. PHA-767491 appears to show anti-proliferative effects against several cancer cell lines, but was also found to target CDK9 and five other kinases as well^110^. Therefore, PHA-76749 likely affects transcription, possibly explaining its pronounced anti-proliferative effects. In contrast, XL-413 appears to act rather on only a few cancer cell lines^155,157^. Crystal structures of CDC7 bound to a 140 aa polypeptide of DBF4 and to XL-413 have been obtained^158,159^ and now offer a structure-guided path for modification of existing compounds to enhance selectivity^149^. Moreover, it is noteworthy that the CDC7-DBF4 binding interface offers the potential for second site targeting, a strategy that so far has not been employed. Also, small molecule-targeted protein degradation (TPD) is an alternative strategy that could be pursued^160^.

Overall, DDK inhibition appears to be a promising strategy to reduce cancer cell proliferation not only as a mono-therapy but also as a combination-therapy together with PARP-inhibition and immune checkpoint inhibition or even together with well-known chemotherapies. With this review, we make the case that DDK should be viewed as genome integrity kinase, and not only as cell cycle kinase required for proliferation. This aspect deserves further exploration, also in the clinic. The TAK-931 studies^103,139,150,151^ showing apparent BRCAness, HR-deficiency and senescence phenotypes are a first step towards exploiting the function of DDK as a genome integrity kinase, but the multitude of DDK functions in replication stress and DNA repair pathways suggest that creative avenues for effective combination therapies could be found in the future.

### Perspectives

Our understanding of DDK has evolved from viewing it as a dedicated replication initiation kinase to recognizing it as a central regulator of chromosome metabolism. Understanding this broader role requires considering DDK from multiple perspectives. With this review, we make a case that DDK should be deemed a genome maintenance kinase that acts at specific locations across chromosomes and with high temporal specificity from S to G2/M phases of the cell cycle. This model raises several important conceptual questions.

First, how is DDK substrate specificity controlled? Structural studies of DDK bound to the Mcm2-7 helicase have revealed detailed docking interactions that enable highly selective phosphorylation. Whether similar docking mechanisms operate for the many additional substrates remains largely unknown. Understanding how DDK selects substrates on chromatin will be essential for dissecting its different cellular functions.

Second, how does DDK activity and or substrate selection change in response to DNA damage and replication stress? Several studies suggest that checkpoint kinases alter DDK regulation under these conditions, potentially redirecting the kinase toward new substrates involved in genome maintenance. Determining whether such substrate switching occurs and how it is controlled will be important for understanding DDK’s role as a genome integrity regulator.

Third, to what extent are DDK functions conserved across organisms? While many aspects of helicase activation appear evolutionarily conserved, other functions—such as specific DNA repair roles—may differ between yeast and vertebrates. Comparative studies across model systems will therefore remain valuable for identifying core versus organism-specific mechanisms.

These questions mark promising starting points for basic biology research, and at the same time, they provide the intellectual foundation for DDK-targeted therapies. Here, promising preclinical data with DDK inhibitors need to be followed up with further advanced compound designs to increase the chances in clinical trials, which in the future can hopefully leverage DDK inhibition to provide new avenues in cancer therapy.

## Supplementary information

Transparent Peer Review file

nr-reporting-summary

Article File
