# Supplementary material for: Exploring the multifaceted Dbf4-dependent kinase from temporal, spatial, and substrate repertoire perspectives
Source: Commun Biol. 2026 Jun 23;9:858. doi: 10.1038/s42003-026-10512-5 (PMC13291351; doi:10.1038/s42003-026-10512-5)
Supplement: Supplementary file 3 — Article File [file 42003_2026_10512_MOESM3_ESM.pdf]

Box 1 – Three perspectives of DDK function:

This review approaches DDK function from three distinct perspectives:

Temporal perspective - DDK activity is tightly linked to the cell cycle. It becomes active at the onset of S phase and remains active through G2 and mitosis before being downregulated by APC/C-dependent degradation of Dbf4. This timing allows DDK to control DNA replication, but also to activate genome integrity pathways that act post-replicatively.

Spatial perspective - DDK acts primarily as a chromosome-associated kinase. It localizes to replication origins, centromeres and replisomes through interactions with chromatin-bound factors. Local enrichment enables selective phosphorylation of proteins involved in DNA replication and chromosome organization, while phosphatases such as Rif1–PP1 counteract DDK activity.

Substrate repertoire perspective - Beyond the replicative helicase Mcm2–7, DDK phosphorylates proteins involved in replication stress responses, DNA repair and chromosome segregation. These substrates position DDK at the interface between DNA replication and genome stability pathways.

Together, these perspectives highlight DDK as a regulator of chromosome metabolism that coordinates DNA replication with genome maintenance across the cell cycle.
