# Supplementary material for: Exploring the multifaceted Dbf4-dependent kinase from temporal, spatial, and substrate repertoire perspectives
Source: Commun Biol. 2026 Jun 23;9:858. doi: 10.1038/s42003-026-10512-5 (PMC13291351; doi:10.1038/s42003-026-10512-5)
Supplement: Supplementary file 1 — Transparent Peer Review file [file 42003_2026_10512_MOESM1_ESM.pdf]

nature portfolio

Peer Review File

Exploring the multifaceted Dbf4-dependent kinase from temporal, spatial, and substrate repertoire perspectives

Corresponding Author: Professor Boris Pfander

This file contains all reviewer reports in order by version, followed by all author rebuttals in order by version.

Version 0:

Reviewer comments:

Reviewer #1

(Remarks to the Author)
General assessment
This manuscript provides a timely and comprehensive review of DDK and its roles in DNA replication and genome stability. The overall structure, examining DDK from temporal, spatial, and functional perspectives, is clear and conceptually appealing. The literature coverage appears broad and up to date, and the review successfully highlights the increasing recognition that DDK functions extend beyond its classical role in replication initiation.

Overall, this is a valuable contribution. However, several aspects of the manuscript could be improved to enhance clarity, accessibility, and conceptual integration. In particular, the review would benefit from stronger pedagogical framing, clearer distinction between organism-specific and conserved mechanisms, improved figure design, and a more integrative discussion of the emerging conceptual framework around DDK function.

Major comment

1. Accessibility and definition of key concepts. The manuscript assumes a relatively high level of prior knowledge and introduces several acronyms and concepts without definition. This may limit accessibility for readers outside the immediate DDK, replication or repair fields. Examples include: BIR, docking, START and even CDK… These terms should be briefly defined at first appearance. Improving this aspect would broaden the audience and make the review more useful for readers from neighboring fields. Related to this point, the introduction could provide a slightly clearer conceptual framework for DNA replication. In particular, briefly explaining the difference between origin licensing and origin firing, as well as the role of the replisome and helicase activation, would help contextualize DDK's central role. A simple schematic illustrating licensing versus firing could also be helpful.

2. Clarification of kinase cooperation and redundancy. One of the strengths of the manuscript is the emphasis on interactions between DDK and other cell-cycle kinases. However, this theme could be developed more explicitly. In particular, the review could further clarify: the nature of DDK–CDK cooperation, situations where phosphorylation events appear sequential versus partially redundant, the potential role of Cdc5/PLK in late mitotic functions. Expanding this discussion would strengthen the conceptual framework linking DDK to broader cell-cycle kinase networks.

3. Interpretation of the Suski et al. study. The manuscript notes that DDK may be dispensable for proliferation in mammalian cells. However, the conclusions of Suski et al. are somewhat more nuanced. Their study suggests functional redundancy between CDC7 and CDK1 during the G1/S transition, such that at least one of these kinases must be present to allow S-phase entry. It may therefore be helpful to clarify this point in the text to avoid giving the impression that DDK is broadly dispensable for proliferation in mammalian systems. A brief clarification of this interpretation would improve conceptual accuracy.

4. Organization of the discussion on origin activation, late origins, and replication timing. Several elements related to origin regulation appear in different parts of the manuscript and could benefit from clearer organization. For example, the statement that late replicating regions require higher DDK activity (based on work such as Jones et al.) currently appears in the replication stress section. This information might fit more naturally in the section discussing origin firing and replication initiation, where the role of DDK in origin activation is first introduced. More generally, it might be helpful to briefly integrate

the concept of replication timing regulation into this discussion. Both Rif1 depletion and DDK perturbation are known to influence replication timing programs, and mentioning this connection could help bridge the spatial and functional perspectives presented in the review.

5. Contextualization of DDK as a “genome integrity kinase”. The manuscript describes the role of DDK in genome integrity pathways as somewhat surprising. While this rhetorical framing is understandable, it might benefit from additional contextualization. Cell cycle kinases as CDKs and PLKs, phosphorylate numerous DNA repair and genome maintenance factors. It may therefore be useful to situate DDK within this broader network of cell-cycle kinases coordinating genome integrity, rather than presenting this feature as entirely unexpected. This would further strengthen the conceptual narrative of the review.

6. Additional mechanistic context for specific phosphorylation events. In several places, specific phosphorylation events are mentioned without further discussion of their potential implications. For example, the manuscript notes that DDK phosphorylates TOP2A at centromeres in human cells. Where available, it would be helpful to briefly discuss the proposed functional implications of such modifications or the hypotheses that have been suggested in the literature. Even brief contextualization would help readers appreciate the significance of these observations.

7.Toward a more integrative conceptual framework. The review provides a very useful summary of the literature but occasionally remains somewhat descriptive. The manuscript would benefit from a slightly more integrative discussion that synthesizes the temporal, spatial, and functional perspectives proposed by the authors. For example, the authors could further elaborate on questions such as: how DDK substrate selection may shift under replication stress, how DDK functions intersect with those of other cell-cycle kinases, which aspects of DDK regulation appear evolutionarily conserved versus organism-specific. Expanding the perspectives section or adding a brief synthesis after the three conceptual perspectives might help articulate this broader framework.

Minor comments
-The term START should be briefly explained for readers unfamiliar with yeast cell-cycle terminology.
-Some sentences could be streamlined for clarity (e.g., the opening sentence describing DDK regulation during the cell cycle).
-Figures could be improved by providing clearer labeling, clearer organism-specific distinctions, and more precise representation of the mechanisms discussed in the text.
-The section title “Antagonism by Rif1-PP1” could be reconsidered for consistency with the naming of other sections. A title such as “Regulation of DDK by Rif1-PP1” might improve the overall structure.
-For next time, page numbering would facilitate review and navigation.

Reviewer #2

(Remarks to the Author)
The role of DDK is an important and timely topic, especially given recent advances in our understanding of its biology and the growing interest in targeting CDC7 in cancer therapy. Galanti and Pfander present an in-depth overview of many key aspects of DDK function, with a particular focus on mechanistic insights. While the manuscript compiles a great deal of relevant and useful information, we believe the review would benefit from several major improvements that would enhance its clarity, structure, and value to the community.

1. The title and abstract frame the review as providing a spatio-temporal-functional perspective. In practice, however, the manuscript is heavily weighted toward functional aspects (~10 pages with multiple sub-sections), whereas temporal and spatial aspects receive much shorter treatment (~2.5 and ~2 pages). This imbalance creates a mismatch between expectations and content. There is nothing inherently wrong with highlighting DDK function, but the structure should reflect the authors’ intended narrative more faithfully.

2. At present, the overarching theme is not clearly stated in the Introduction, and the flow of ideas can feel disjointed despite the wealth of information provided. The Introduction would be stronger if it clearly outlined what is coming in the following sections, explained the rationale for the chosen structure, and articulated why DDK is such an important target both biologically and clinically. This would help orient readers and anchor the subsequent discussion.

3. The use of phrases such as “(see below)” without referencing a specific section can be confusing, especially when the relevant information appears much later in the text. Consider adding additional subsections or using explicit section titles to guide readers more effectively.

4. In the “spatial” section, Fig. 2 could be improved by using standard panel labels (e.g., a, b, c), or by consistently referring to “left,” “center,” and “right” in the text as it is in the legend. In addition, the statement that “similar to CDK, DDK localizes to the cytoplasm and nucleus..” should be clarified and supported. As written, it also appears inconsistent with the left panel of Figure 2 as well as in a portion of text in the next section (see below).

5. In the “functional” section, the authors describe DDK’s role in genome integrity as “surprising,” yet this section makes up a major portion of the review and describes a function that is now well established. If the intent is to highlight that this role was unexpected historically, that context should be made explicit.

6. The term “predestined,” used to describe how DDK’s chromosomal localization relates to its function, also needs clarification in a scientific context. Furthermore, the remark about a “bias” introduced by the number of studies in the genome-integrity field is unclear, especially regarding how this bias relates specifically to DDK.

7. In the part “DDK and DNA replication stress” (sub-section of “functional”?), the text introduces three major functions of DDK during replication stress but Fig. 3 does not reflect these points. Because these functions are important, separating them better or marking them more distinctly within the text would help readers navigate the material.

8. The “DDK and DNA double-strand break repair” portion is generally well written and clear. The first sentence, however, does not seem essential and could be removed to improve the flow.

9. Given the importance of the Rif1–PP1 regulatory axis, this topic deserves its own sub-section, which is present in the manuscript. The phrasing that DDK functions are regulated by “equilibrium with” Rif1–PP1 could be refined for clarity. Of note, the manuscript alternates between “dephosphorylation” and “de-phosphorylation”; using one spelling consistently would improve readability.

10. The introduction to the clinical relevance of DDK inhibition is brief, and the section would benefit from a more comprehensive overview of available tool compounds, clinical candidates, and the status of ongoing or completed trials (Table?). In addition to noting the completion of the phase II study of TAK-931, it would be important to mention that Takeda Pharmaceuticals has discontinued its CDC7 program due to insufficient clinical efficacy. Similarly, the termination of Schrödinger’s SGR-2921 program following two patient deaths is highly relevant. Potential therapeutic window challenges could be discussed.

11. The mention of DDK-inhibition-induced senescence could also be expanded, for example by citing the important work of Wang et al. (PMID: 31578521).

Overall, this is an interesting and important area of research, and the authors have gathered a substantial amount of relevant information. With improvements to structure, clarity, and thematic consistency, the manuscript has the potential to become a highly useful and authoritative resource for researchers working on DDK biology and its clinical relevance.

Reviewer #3

(Remarks to the Author)
I co-reviewed this manuscript with one of the reviewers who provided the listed reports. This is part of the Communications Biology initiative to facilitate training in peer review and to provide appropriate recognition for Early Career Researchers who co-review manuscripts.

Reviewer #4

(Remarks to the Author)
In this manuscript, the authors provide an extensive overview of the literature related to DDK across several organisms, with particular emphasis on budding yeast, Xenopus, and human cells.

The strengths of the review include: (1) highlighting the multiple functions of DDK in genome stability, and (2) integrating important old and almost forgotten groundbreaking findings with more recent work.

The review appears to be organized around three main themes—temporal regulation, cellular localization, and functional roles—but subsequently expands into largely independent sections, including a discussion of DDK inhibitors and their therapeutic potential in cancer. In its current form, this structure does not seem to work optimally, as several concepts are reiterated across sections

A more focused approach and sharper structure, possibly focusing on the insight from from model organisms, which represents the strength of the manuscript, could be beneficial.

Major comments:

1) The authors should discuss the role of CDC7 in Scc2–Scc4 recruitment to chromatin in Xenopus and SSC2/4 interactions with CDC7/DRF1 as reported by Takahashi et al Genes Dev. 2008 Jul 15;22(14):1894–1905. doi: 10.1101/gad.1683308

2) The review would benefit from inclusion of the early finding that DBF4 localizes to replication origins in budding yeast, identified using a one-hybrid system (Dowell et al., Science 1994 Aug 26;265(5176):1243–1246; doi:10.1126/science.806646).

3) The manuscript refers to a stable DDK–Cdc5 complex in yeast, What biochemical data support this interaction, and can these data be discussed explicitly?

4) It is unclear why the authors state that the studies by Rainey (2020) and Jones (2021) are contradictory. The arguments presented appear to address distinct biological processes—replication initiation versus fork reversal—rather than opposing conclusions.

6) The question of whether DDK is essential for replication and DNA replication is controversial and central to understanding the effects of DDK inhibitors. It should be discussed with attention. In this reviewer's opinion DDK is essential for replication in human cells, however cells display a wide range of tolerance to reduced levels of origin activation. Few points below:

a) CDC7 is classified as a core essential gene in human cells (DepMap.org), and loss of CDC7 impairs proliferation in all tested cell types (>1000).
b) The potency of different DDKi greatly vary in in vitro kinase assays and proportionally in cell-based assay. When used at appropriate concentrations, all tested cell lines are sensitive to specific DDK inhibitors (including XL413).

c) DDK inhibition blocks DNA replication in the Xenopus cell-free extract system (Poh et al., Open Biol. 2013; doi:10.1098/rsob.130138). The reference currently cited as "Prokhorova et al." should be carefully checked.

7) No CDC7- PROTAC has yet been disclosed and the compound cited (Ji et al., 2024) is a CDK7 degrader rather than a CDC7 degrader.

8) The final section ("Perspectives") would benefit from substantially greater focus and clarity.

Version 1:

Reviewer comments:

Reviewer #1

(Remarks to the Author)
The revised manuscript presents a comprehensive and well-structured review of the functions of DDK, expanding its role from a canonical replication initiation kinase to a broader regulator of genome integrity. The main claims are clearly articulated and timely, and the synthesis of recent literature will be of strong interest to researchers working in DNA replication, replication stress, and DNA repair.

The revised version has significantly improved in clarity, balance, and conceptual framing. In particular, the authors now better contextualize the role of DDK in genome integrity pathways.

The section on Rif1-PP1 provides a clearer and more integrated view of the antagonistic relationship with DDK, and its implications beyond replication initiation are now well developed. Additionally, I like the inclusion of a dedicated perspectives section, as it outlines key open questions and helps frame the field in a more integrative manner.

Overall, the manuscript is convincing as a review article. In its current form, I believe the manuscript will be a valuable resource for the community and has the potential to influence how DDK is conceptualized within the broader context of genome maintenance.

Reviewer #2

(Remarks to the Author)
Thank you for addressing the comments. This comprehensive review will serve the DDK community as well as the greater field of researchers studying cell biology.

Reviewer #3

(Remarks to the Author)
I co-reviewed this manuscript with one of the reviewers who provided the listed reports. This is part of the Communications Biology initiative to facilitate training in peer review and to provide appropriate recognition for Early Career Researchers who co-review manuscripts.

Reviewer #4

(Remarks to the Author)
The authors have addressed all the comments in a constructive manner, and the revised manuscript is substantially improved in clarity, precision and overall flow. I support its publication.
I have one minor point for consideration:
Line 605: The term "creative" drug design is ambiguous, and its relevance to improving DDKi therapy is therefore unclear. I would recommend clarifying/expanding on what is required for obtaining an improvement or revising the sentence.

Open Access This Peer Review File is licensed under a Creative Commons Attribution 4.0 International License, which permits use, sharing, adaptation, distribution and reproduction in any medium or format, as long as you give appropriate credit to the original author(s) and the source, provide a link to the Creative Commons license, and indicate if changes were made.
In cases where reviewers are anonymous, credit should be given to 'Anonymous Referee' and the source.
The images or other third party material in this Peer Review File are included in the article's Creative Commons license, unless indicated otherwise in a credit line to the material. If material is not included in the article's Creative Commons license and your intended use is not permitted by statutory regulation or exceeds the permitted use, you will need to obtain permission directly from the copyright holder.
To view a copy of this license, visit https://creativecommons.org/licenses/by/4.0/

Point-by-point response:

We thank the reviewers for their careful and thoughtful evaluation of our manuscript. We were particularly encouraged that the reviewers recognized the review as **timely and comprehensive**, noted that it **covers the relevant literature broadly and up-to-date**, and highlighted the **conceptual value of examining DDK from temporal, spatial, and functional perspectives**.

At the same time, we are grateful for the many constructive suggestions aimed at improving clarity, accessibility, and conceptual integration. We have carefully revised the manuscript to address these points, including , strengthening the conceptual narrative, improving accessibility for the non-expert reader, clarifying organism-specific versus conserved mechanisms, and refining the figures. Below we respond to each comment in detail.

Reviewers' comments:

Reviewer #1 (Remarks to the Author):

General assessment
This manuscript provides a timely and comprehensive review of DDK and its roles in DNA replication and genome stability. The overall structure, examining DDK from temporal, spatial, and functional perspectives, is clear and conceptually appealing. The literature coverage appears broad and up to date, and the review successfully highlights the increasing recognition that DDK functions extend beyond its classical role in replication initiation.

Overall, this is a valuable contribution. However, several aspects of the manuscript could be improved to enhance clarity, accessibility, and conceptual integration. In particular, the review would benefit from stronger pedagogical framing, clearer distinction between organism-specific and conserved mechanisms, improved figure design, and a more integrative discussion of the emerging conceptual framework around DDK function.

Response: We thank the reviewer for the positive assessment and for the constructive criticism, which has greatly helped us to improve our manuscript.

Major comment

1. Accessibility and definition of key concepts. The manuscript assumes a relatively high level of prior knowledge and introduces several acronyms and concepts without definition. This may limit accessibility for readers outside the immediate DDK, replication or repair fields. Examples include: BIR, docking, START and even CDK… These terms should be briefly defined at first appearance. Improving this aspect would broaden the audience and make the review more useful for readers from neighboring fields. Related to this point, the introduction could provide a slightly clearer conceptual framework for DNA replication. In particular, briefly explaining the difference between origin licensing and origin firing, as well as the role of the replisome and helicase activation, would help contextualize DDK's central role. A simple schematic illustrating licensing versus firing could also be helpful.

Response: We thank the reviewer for this helpful suggestion. We agree that improving accessibility will broaden the readership of the review. We have therefore revised the Introduction and several sections of the manuscript to define key concepts at their first appearance, including BIR, START, CDK, and the concept of substrate docking. In addition, we added a short explanatory paragraph describing the two-tiered temporal regulation of DNA replication with origin licensing and origin firing, and how DDK specifically acts during origin firing. We have also included licensing and firing in

Fig.1. These additions provide a clearer conceptual framework for understanding the role of DDK in replication initiation.

Changes in text: "Replication initiation is a two-step process, during which the replicative helicase is first loaded at origins of replication in the form of an inactive precursor (origin licensing, Fig. 1b). In S-phase, these helicase precursors become activated by phosphorylation and the association of accessory subunits (origin firing, (Fig. 1b). Specifically, DDK functions during origin firing: it targets the catalytic Mcm2-7 core of the CMG helicase to alleviate auto-inhibition (Cheng et al., 2022; Greiwe et al., 2022; Saleh et al., 2022; Sheu and Stillman, 2010) and generate binding sites for Sld3, a key origin firing factor (Deegan et al., 2016).

2. Clarification of kinase cooperation and redundancy. One of the strengths of the manuscript is the emphasis on interactions between DDK and other cell-cycle kinases. However, this theme could be developed more explicitly. In particular, the review could further clarify: the nature of DDK–CDK cooperation, situations where phosphorylation events appear sequential versus partially redundant, the potential role of Cdc5/PLK in late mitotic functions. Expanding this discussion would strengthen the conceptual framework linking DDK to broader cell-cycle kinase networks.

Response: We thank the reviewer for highlighting this important conceptual aspect. We agree that cooperation with other cell cycle kinases is one of the most fascinating aspects of DDK biology. We have therefore expanded the chapter on "DDK – a cell cycle kinase" to include this aspect and name the known examples of substrates that are phosphorylated by DDK and CDK, as well as by DDK and Cdc5 and specifically highlight those where CDK appears to prime for DDK phosphorylation.

Changes in text: "Notably, CDK and DDK are not only active at the same time, but sometimes even phosphorylate the same substrates. One example is given by the regulation of homologous recombination in budding yeast (see "DDK and DNA double-strand break repair"), which was known for some time to be under CDK control (Chen et al., 2011; Huertas et al., 2008; Ira et al., 2004), but according to our recent data is under dual kinase control by CDK and DDK (Galanti et al., 2024). In some cases, CDK and DDK even target the same substrate interdependently and CDK catalyses a priming phosphorylation which is extended by DDK for a secondary phosphorylation step (examples include Dna2, Eco1, Mcm4/6, Mer2, Sld2 in budding yeast (Galanti et al., 2024; Lyons et al., 2013; Randell et al., 2010; Reusswig et al., 2016; Sasanuma et al., 2008; Wan et al., 2008)). Further work will need to clarify whether DDK should be viewed as a cell cycle kinase that acts primarily in conjunction with CDK ("dual kinase phosphorylation").

Additionally, several proteins are known in the budding yeast system which are simultaneously phosphorylated by DDK and Cdc5. This group of proteins include Lrs4, Mms4, Rec8 and Sld2 (Katis et al., 2010; Matos et al., 2008; Princz et al., 2017; Reusswig et al., 2016), but priming phosphorylation or interdependency has not been observed for any of these substrates. These data therefore suggest that these phosphorylation events have independent functions, even though the kinases interact.."

3. Interpretation of the Suski et al. study. The manuscript notes that DDK may be dispensable for proliferation in mammalian cells. However, the conclusions of Suski et al. are somewhat more nuanced. Their study suggests functional redundancy between CDC7 and CDK1 during the G1/S transition, such that at least one of these kinases must be present to allow S-phase entry. It may therefore be helpful to clarify this point in the text to avoid giving the impression that DDK is broadly dispensable for proliferation in mammalian systems. A brief clarification of this interpretation would improve conceptual accuracy.

Response: We thank the reviewer for pointing this out. We have revised the relevant paragraph to clarify that Suski et al. propose functional redundancy between CDC7 and CDK1 during the G1/S transition rather than complete dispensability of DDK.

New text:

"Of note, while both DDK and CDK are essential for DNA replication in yeast and Xenopus replication systems (Hardy et al., 1997; Poh et al., 2014; Sheu and Stillman, 2010), a recent study suggests that DDK is not strictly required for proliferation in non-transformed human and mouse cells (Suski et al., 2022). The authors used different systems (analog-sensitive kinase alleles, auxin-inducible degrons as well as knock-outs) to interfere with DDK activity/stability and observed proliferation in different non-cancerous human cell lines and mice tissues despite a strong decrease or even absence of DDK activity, suggesting that DDK can be dispensable for proliferation. Notably, the same study found Cyclin-B CDK1, known to be mainly active in late S-phase/mitosis to act redundantly with DDK for the initiation of DNA replication, and lack of both CDK1 and DDK was found to be lethal (Suski et al., 2022). This finding suggests functional redundancy of the two cell cycle kinases and that – despite not strictly essential – DDK is an important driver of proliferation."

4. Organization of the discussion on origin activation, late origins, and replication timing. Several elements related to origin regulation appear in different parts of the manuscript and could benefit from clearer organization. For example, the statement that late replicating regions require higher DDK activity (based on work such as Jones et al.) currently appears in the replication stress section. This information might fit more naturally in the section discussing origin firing and replication initiation, where the role of DDK in origin activation is first introduced. More generally, it might be helpful to briefly integrate the concept of replication timing regulation into this discussion. Both Rif1 depletion and DDK perturbation are known to influence replication timing programs, and mentioning this connection could help bridge the spatial and functional perspectives presented in the review.

Response: We agree with the reviewer that this point fits more naturally in the replication initiation discussion. We have therefore moved the relevant discussion to the DNA replication section and expanded it to introduce the concept of replication timing regulation and DDKs potential roles therein.

Changes in text: "DNA replication follows a temporal program, with individual replication origins becoming activated with specific timing during S phase. Interestingly, DDK may be involved in generating this temporal program. In budding yeast, DDK was found to be a limiting factor for DNA replication, which was proposed to associate first with early replicating origins and subsequently be recycled to late replicating origins (Mantiero et al., 2011). A similar phenomenon was also observed in human cells, where CDC7-analog sensitive cell lines revealed that late replicating regions required higher DDK activity compared to early replicating regions (Jones et al., 2021)."

5. Contextualization of DDK as a "genome integrity kinase". The manuscript describes the role of DDK in genome integrity pathways as somewhat surprising. While this rhetorical framing is understandable, it might benefit from additional contextualization. Cell cycle kinases as CDKs and PLKs, phosphorylate numerous DNA repair and genome maintenance factors. It may therefore be useful to situate DDK within this broader network of cell-cycle kinases coordinating genome integrity, rather than presenting this feature as entirely unexpected. This would further strengthen the conceptual narrative of the review.

Response: We agree. Our original intention was to highlight the historical perspective that DDK's functions in genome integrity pathways were initially surprising as it was viewed to be a cell cycle kinase regulating S phase and DNA replication. With current knowledge of genome integrity proteins being major substrates also for other cell cycle kinases, this is no longer surprising. Consistent with the suggestion of reviewer #2, we have toned this section down and now provide a more neutral statement.

Changes in text: "Next to its temporal and spatial regulation, we can also approach DDK function by focussing on its phosphorylation substrates. Many DDK targets have established roles in genome integrity. We will therefore focus on the perspective of DDK as a genome integrity kinase."

6. Additional mechanistic context for specific phosphorylation events. In several places, specific phosphorylation events are mentioned without further discussion of their potential implications. For example, the manuscript notes that DDK phosphorylates TOP2A at centromeres in human cells. Where available, it would be helpful to briefly discuss the proposed functional implications of such modifications or the hypotheses that have been suggested in the literature. Even brief contextualization would help readers appreciate the significance of these observations.

Response: We thank the reviewer for the helpful suggestion. Following this comment, we clarified functional implications of DDK-mediated phosphorylation for TOP2A. We also carefully checked that we have given sufficient detail on the functional implications of the phosphorylation marks for the other substrates as well.

Changes in text: "Also in human cells DDK acts at centromeres, where it leads to phosphorylation of TOP2A and mediates release of TOP2A from centromeres (Wu et al., 2016)

7.Toward a more integrative conceptual framework. The review provides a very useful summary of the literature but occasionally remains somewhat descriptive. The manuscript would benefit from a slightly more integrative discussion that synthesizes the temporal, spatial, and functional perspectives proposed by the authors. For example, the authors could further elaborate on questions such as: how DDK substrate selection may shift under replication stress, how DDK functions intersect with those of other cell-cycle kinases, which aspects of DDK regulation appear evolutionarily conserved versus organism-specific. Expanding the perspectives section or adding a brief synthesis after the three conceptual perspectives might help articulate this broader framework.

Response: We agree with the reviewer that these are important and exciting questions of DDK biology and we very much hope that future research will clarify them. At the moment, we feel that we do not have definitive answers and have therefore included these aspects in our revised version of the perspective chapter at the end of the review, which we have reshaped in response to this point.

Changes in text: "Our understanding of DDK has evolved from viewing it as a dedicated replication initiation kinase to recognizing it as a central regulator of chromosome metabolism. Understanding this broader role requires considering DDK from multiple perspectives. With this review, we make a case that DDK should be deemed a genome maintenance kinase that acts at specific locations across chromosomes and with high temporal specificity from S to G2/M phase of the cell cycle. This model raises several important conceptual questions.

First, how is DDK substrate specificity controlled? Structural studies of DDK bound to the Mcm2-7 helicase have revealed detailed docking interactions that enable highly selective phosphorylation. Whether similar docking mechanisms operate for the many additional substrates remains largely unknown. Understanding how DDK selects substrates on chromatin will be essential for dissecting its different cellular functions.

Second, how does DDK activity and or substrate selection change in response to DNA damage and replication stress? Several studies suggest that checkpoint kinases alter DDK regulation under these conditions, potentially redirecting the kinase toward new substrates involved in genome maintenance. Determining whether such substrate switching occurs and how it is controlled will be important for understanding DDK's role as a genome integrity regulator.

Third, to what extent are DDK functions conserved across organisms? While many aspects of helicase activation appear evolutionarily conserved, other functions—such as specific DNA repair roles—may differ between yeast and vertebrates. Comparative studies across model systems will therefore remain valuable for identifying core versus organism specific mechanisms.

These questions mark promising starting points for basic biology research, at the same time they provide the intellectual fundament for DDK-targeted therapies. Here, promising preclinical data with

DDK inhibitors need to be followed up with further advanced compound designs to increase the chances in clinical trials, which in the future can hopefully leverage DDK inhibition to provide new avenues in cancer therapy."

Minor comments

-The term START should be briefly explained for readers unfamiliar with yeast cell-cycle terminology.

Response: We have added a brief definition of START when the term first appears.

-Some sentences could be streamlined for clarity (e.g., the opening sentence describing DDK regulation during the cell cycle).

Response: We have worked on the formulation of the opening sentence for DDK cell cycle-regulation as well as on several other places of the manuscript.

-Figures could be improved by providing clearer labeling, clearer organism-specific distinctions, and more precise representation of the mechanisms discussed in the text.

Response: We have worked on the clarity of figure presentation, for example by introducing panel labelling for Figure 2, or by providing a revised versions of Figure 1 and 3, which now matches better to our text by showing DDK replication stress functions more broadly.

-The section title "Antagonism by Rif1-PP1" could be reconsidered for consistency with the naming of other sections. A title such as "Regulation of DDK by Rif1-PP1" might improve the overall structure.

Response: We have changed the title to "Regulation of DDK substrates by Rif-PP1", similar to what was suggested by the reviewer.

-For next time, page numbering would facilitate review and navigation.

Response: We apologize and have added page numbering to the revised version of the manuscript.

Reviewer #2 (Remarks to the Author):

The role of DDK is an important and timely topic, especially given recent advances in our understanding of its biology and the growing interest in targeting CDC7 in cancer therapy. Galanti and Pfander present an in-depth overview of many key aspects of DDK function, with a particular focus on mechanistic insights. While the manuscript compiles a great deal of relevant and useful information, we believe the review would benefit from several major improvements that would enhance its clarity, structure, and value to the community.

Response: We thank the reviewers for their positive assessment and thoughtful comments. We addressed their constructive criticism and feel that me can now present a significantly improved manuscript.

1. The title and abstract frame the review as providing a spatio-temporal-functional perspective. In practice, however, the manuscript is heavily weighted toward functional aspects (~10 pages with multiple sub-sections), whereas temporal and spatial aspects receive much shorter treatment (~2.5 and ~2 pages). This imbalance creates a mismatch between expectations and content. There is nothing inherently wrong with highlighting DDK function, but the structure should reflect the authors' intended narrative more faithfully.

Response: We thank the reviewer for this insightful comment, which highlights that a key part of our manuscript lacked clarity and may thereby have led to a misconception. Our intention was to use the temporal, spatial and substrate sections as conceptual entry points that frame the broader discussion of DDK function. For this, we wanted to utilize the knowledge on temporal regulation ("A cell cycle kinase"), spatial localization ("A chromosomal kinase") and phosphorylation substrates ("A genome integrity kinase") to each give a different perspective of DDK function. We agree that the current structure places greater emphasis on substrates and function than on temporal and spatial regulation. To make this clearer, we have revised the introduction to explicitly explain the rationale for this structure.

Changes in manuscript "Introduction: The Dbf4-dependent kinase Cdc7 (DDK) is an essential cell cycle kinase, conserved throughout eukaryotes. DDK is active as a heterodimer, consisting of the S/T kinase Cdc7 and the regulatory subunit Dbf4. DDK is most well-known as central regulator of DNA replication, but recent work suggests a more widespread function in genome maintenance. In this review, we discuss DDK function from three complementary perspectives. First, we consider the temporal regulation of DDK during the cell cycle and discuss DDK as a cell cycle kinase. Second, we examine how subcellular and chromosomal localization, as well as docking to substrates and substrate complexes contributes to substrate selection and discuss DDK as a chromosomal kinase. Finally, we summarize current knowledge on DDK substrates and discuss the emerging view of DDK as a genome integrity kinase. Together, these perspectives highlight DDK as a multifunctional kinase whose temporal regulation, chromosomal localization, and substrate repertoire collectively coordinate cell cycle progression with the maintenance of genome integrity."

2. At present, the overarching theme is not clearly stated in the Introduction, and the flow of ideas can feel disjointed despite the wealth of information provided. The Introduction would be stronger if it clearly outlined what is coming in the following sections, explained the rationale for the chosen structure, and articulated why DDK is such an important target both biologically and clinically. This would help orient readers and anchor the subsequent discussion.

Response: We agree with the reviewer that the introduction could more clearly outline structure and central theme of the review and its focus on the three different perspectives of DDK function. As discussed in point 1, we now included this concept at the very beginning of the Introduction.

3. The use of phrases such as "(see below)" without referencing a specific section can be confusing, especially when the relevant information appears much later in the text. Consider adding additional subsections or using explicit section titles to guide readers more effectively.

Response: Agreed, we have now referenced the specific sub-sections, whenever we refer to a different part of the manuscript.

4. In the "spatial" section, Fig. 2 could be improved by using standard panel labels (e.g., a, b, c), or by consistently referring to "left," "center," and "right" in the text as it is in the legend. In addition, the statement that "similar to CDK, DDK localizes to the cytoplasm and nucleus.." should be clarified and supported. As written, it also appears inconsistent with the left panel of Figure 2 as well as in a portion of text in the next section (see below).

Response: We thank the reviewer for this suggestion. We agree that the statement regarding DDK localization needed clarification, which we have now included in the revised version of the manuscript. We also realized that we needed additional references to support the findings from human cells as well. Furthermore, we have revised Figure 2 to include standard panel labels (Fig. 2a–c) and updated the text accordingly to reference these panels.

Changes to text: "A second key attribute of DDK is its sub-cellular localization. In yeast and human cells DDK localizes predominantly to the nucleus (Fig. 2a, Huh et al., 2003; Jiang, 1999b; Jiang and Hunter, 1997; Kumagai et al., 1999; Sato, 1997; Wu et al., 2016; Yoon and Campbell, 1991). Additionally, using biochemical fractionation, DDK was found to specifically localize to chromatin in yeast cells and Xenopus egg extracts (Bansal et al., 2026; Jares et al., 2004; Jares and Blow, 2000; Natsume et al., 2013; Weinreich, 1999; Yanow et al., 2003)."

5. In the "functional" section, the authors describe DDK's role in genome integrity as "surprising," yet this section makes up a major portion of the review and describes a function that is now well established. If the intent is to highlight that this role was unexpected historically, that context should be made explicit.

Response: We agree, this point was also raised by Reviewer #1. Our original intention was to highlight the historical perspective that DDK's functions in genome integrity pathways were surprising as it was viewed to be a cell cycle kinase regulating S phase and DNA replication. With current knowledge of genome integrity proteins being major substrates also for other cell cycle kinases, this is no longer surprising. We have therefore toned this section down and provide a more balanced view.

Changes in manuscript: "Next to its temporal and spatial regulation, we can also approach DDK function by focussing on its phosphorylation substrates. Many DDK targets have established roles in genome integrity. We will therefore focus on the perspective of DDK as a genome integrity kinase."

6. The term "predestined," used to describe how DDK's chromosomal localization relates to its function, also needs clarification in a scientific context. Furthermore, the remark about a "bias" introduced by the number of studies in the genome-integrity field is unclear, especially regarding how this bias relates specifically to DDK.

Response: We agree that this wording was imprecise in both cases and have replaced it with a more neutral scientific formulation and removed the other sentence ("bias") altogether.

Changes in manuscript: "Given its cell cycle regulation and chromatin association, DDK is well positioned to regulate genome integrity pathways."

7. In the part "DDK and DNA replication stress" (sub-section of "functional"?), the text introduces three major functions of DDK during replication stress but Fig. 3 does not reflect these points. Because these functions are important, separating them better or marking them more distinctly within the text would help readers navigate the material.

Response: We thank the reviewer for identifying this inconsistency. We have revised Figure 3 to explicitly illustrate the passive and active replication stress functions described in the text: inhibition of origin firing, regulation of stalled replication forks, and modulation of checkpoint signalling.

8. The “DDK and DNA double-strand break repair” portion is generally well written and clear. The first sentence, however, does not seem essential and could be removed to improve the flow.

Response: Agreed, we have removed the sentence.

9. Given the importance of the Rif1–PP1 regulatory axis, this topic deserves its own sub-section, which is present in the manuscript. The phrasing that DDK functions are regulated by “equilibrium with” Rif1–PP1 could be refined for clarity. Of note, the manuscript alternates between “dephosphorylation” and “de-phosphorylation”; using one spelling consistently would improve readability.

Response: Agreed, we have rewritten this sentence for clarity and now use consistently dephosphorylation throughout the manuscript.

Changes in manuscript: “The aforementioned functions of DDK are counteracted by dephosphorylation mediated by the Rif1–PP1 phosphatase complex.”

10. The introduction to the clinical relevance of DDK inhibition is brief, and the section would benefit from a more comprehensive overview of available tool compounds, clinical candidates, and the status of ongoing or completed trials (Table?). In addition to noting the completion of the phase II study of TAK-931, it would be important to mention that Takeda Pharmaceuticals has discontinued its CDC7 program due to insufficient clinical efficacy. Similarly, the termination of Schrödinger’s SGR-2921 program following two patient deaths is highly relevant. Potential therapeutic window challenges could be discussed.

Response: We thank the reviewer for this suggestion and have now included a discussion about the discontinuation of the TAK-931 program, the incidents related to SGR2921 and the potential need in the development of new drugs in our manuscript. We decided against including a table of compounds and clinical trials, as this would shift the focus away from DDK biology to the pharmacology of DDK inhibitors. We refer our readers to last year’s review by Peng et al. (doi.org/10.1016/j.ejmech.2025.117491), which provides a comprehensive overview on compounds and the corresponding clinical trials.

Changes in manuscript: “These findings suggest a therapeutic window could be found for DDK inhibitors, where overall toxicity and side-effects are minimal. In the specific case of TAK-931 a phase I and II clinical trial suggested a good safety profile (Kuboki et al., 2022), but the corresponding program has been discontinued at Takeda Pharmaceuticals. Furthermore, a phase I clinical trial of an unrelated compound called SGR2921 (Schrödinger) has been discontinued following Serious Adverse Events. Therefore, creative drug design is likely important for the development of DDK-inhibition as a therapy.”

11. The mention of DDK-inhibition-induced senescence could also be expanded, for example by citing the important work of Wang et al. (PMID: 31578521).

Response: We thank the reviewer for this comment and apologize for the oversight of not including the reference to Wang et al. paper. We expanded this section and included the reference.

Changes in manuscript: “Moreover, DDK inhibition by XL413 or TAK-931 induces a senescence phenotype that triggered inflammatory responses in cell-culture-based cancer cell models, as well as a myeloma mouse model (Cazzaniga et al., 2024; Morita et al., 2023; Wang et al., 2019). Consistently, DDK inhibition was found to act synergistically with immune checkpoint inhibitors (anti-mPD-1 and anti-CTLA-4 specifically, (Morita et al., 2023)). Moreover, XL413 showed synergism with anti-PD-1, as well as mTOR inhibitors in mouse xenograft models for hepatocellular carcinoma (Wang et al., 2019;

Zhang et al., 2023). DDK inhibition therefore appears to be also promising in combination with mTOR inhibition or immune checkpoint therapy."

Overall, this is an interesting and important area of research, and the authors have gathered a substantial amount of relevant information. With improvements to structure, clarity, and thematic consistency, the manuscript has the potential to become a highly useful and authoritative resource for researchers working on DDK biology and its clinical relevance.

Reviewer #3 (Remarks to the Author):

I co-reviewed this manuscript with one of the reviewers who provided the listed reports. This is part of the Communications Biology initiative to facilitate training in peer review and to provide appropriate recognition for Early Career Researchers who co-review manuscripts.

Reviewer #4 (Remarks to the Author):

In this manuscript, the authors provide an extensive overview of the literature related to DDK across several organisms, with particular emphasis on budding yeast, Xenopus, and human cells.

The strengths of the review include: (1) highlighting the multiple functions of DDK in genome stability, and (2) integrating important old and almost forgotten groundbreaking findings with more recent work.

The review appears to be organized around three main themes—temporal regulation, cellular localization, and functional roles—but subsequently expands into largely independent sections, including a discussion of DDK inhibitors and their therapeutic potential in cancer. In its current form, this structure does not seem to work optimally, as several concepts are reiterated across sections

A more focused approach and sharper structure, possibly focusing on the insight from from model organisms, which represents the strength of the manuscript, could be beneficial.

Response: We thank the reviewer for their constructive criticism. We have now addressed their comments in full and hope they share our assessment that we now provide a strongly improved manuscript.

Major comments:

1) The authors should discuss the role of CDC7 in Scc2–Scc4 recruitment to chromatin in Xenopus and SSC2/4 interactions with CDC7/DRF1 as reported by Takahashi et al Genes Dev. 2008 Jul 15;22(14):1894–1905. doi: 10.1101/gad.1683308

Response: We thank the reviewer for drawing our attention to this important study. We have now included a discussion of CDC7–DRF1 interactions with cohesin loading factors and the implications for chromosome organization in the section where we discuss DDK as a chromosomal kinase.

Changes in manuscript: “DDK’s role as a chromosomal kinase is underscored by its phosphorylation of key chromosome architectural proteins. Substrates include histone chaperones and chromatin remodellers, such as human CAF1 and budding yeast INO80, both of which undergo DDK-dependent regulation during DNA replication (Bansal et al., 2026; Gérard et al., 2006). Furthermore, DDK was found to regulate the cohesin complex in different organisms, even though it appears to be acting on distinct proteins. In budding yeast, DDK phosphorylates the cohesion establishment factor Eco1 creating a cell-cycle-dependent degron, which mediates S-phase specificity of Eco1 function (Lyons et al., 2013; Seoane and Morgan, 2017). DDK also phosphorylates Rec8, the [illegible]-kleisin subunit of the meiosis-specific cohesin variant, and promotes its cleavage by separase (Katis et al., 2010). In the Xenopus egg extract system and also human cells, DDK was shown to act on the cohesin loader Scc2-Scc4 and promote cohesin loading, suggesting a role in establishing chromosome architecture (Takahashi et al., 2008; Zheng et al., 2018). Lastly, the cohesion regulator Pds5b is phosphorylated by DDK as well, but this mechanism still awaits detailed functional characterization (Jones et al., 2021).”

2) The review would benefit from inclusion of the early finding that DBF4 localizes to replication origins in budding yeast, identified using a one-hybrid system (Dowell et al., Science 1994 Aug 26;265(5176):1243–1246; doi:10.1126/science.806646).

Response: We thank the reviewer for highlighting this important early study. We have now incorporated this work into the spatial section when discussing origin localization of DDK.

Changes in manuscript: "A second key attribute of DDK is its sub-cellular localization. In yeast and human cells DDK localizes predominantly to the nucleus (Fig. 2a, Huh et al., 2003; Jiang, 1999b; Jiang and Hunter, 1997; Kumagai et al., 1999; Sato, 1997; Wu et al., 2016; Yoon and Campbell, 1991). Additionally, using biochemical fractionation, DDK was found to specifically localize to chromatin in yeast cells and Xenopus egg extracts (Bansal et al., 2026; Jares et al., 2004; Jares and Blow, 2000; Natsume et al., 2013; Weinreich, 1999; Yanow et al., 2003). Moreover, DDK chromatin binding even correlates with its DNA replication initiation function as DDK was shown to preferentially localize to early-firing origins in yeast and localization to origins coincided with the timing of their activation (Dowell et al., 1994; Fang et al., 2017; Natsume et al., 2013). Interaction with Mcm2-7 proteins is likely contributing to recruitment to replication origins (Francis et al., 2009; Jares and Blow, 2000; Ramer et al., 2013; Sheu and Stillman, 2006; Weinreich, 1999). Furthermore, an interaction with the fork progression complex observed in both budding and fission yeast suggests that DDK is also recruited to replisomes (see DDK and DNA replication stress: Matsumoto et al., 2005; Murakami and Keeney, 2014a; Shimmoto et al., 2009)."

3) The manuscript refers to a stable DDK–Cdc5 complex in yeast, What biochemical data support this interaction, and can these data be discussed explicitly?

Response: Agreed, there is no data published in a scientific journal that suggest the formation of a stable complex. We have now toned the corresponding passage of the manuscript. Nonetheless, we think it is important to consider that DDK and Cdc5 may have common substrates in M-phase.

Changes in manuscript: "Notably, some of these late functions depend on DDK-binding to the mitotic cell cycle kinase Cdc5 in yeast (Chen and Weinreich, 2010; Hinshaw et al., 2023; Matos et al., 2008; Miller et al., 2009; Princz et al., 2017). It is plausible that DDK may recognize a distinct set of substrates when bound to Cdc5 (Hinshaw et al., 2023; Matos et al., 2008; Princz et al., 2017). These data are therefore consistent with a model whereby DDK phosphorylates two pools of substrates: the first pool contains substrates such as the Mcm2-7 proteins, which are phosphorylated from S- to M-phase and depend specifically on DDK, while the second pool of substrates is phosphorylated specifically in M-phase and depend on DDK, but potentially also Cdc5. Currently, it is unclear whether physical interaction of DDK and Cdc5 is unique to budding yeast or evolutionarily conserved."

4) It is unclear why the authors state that the studies by Rainey (2020) and Jones (2021) are contradictory. The arguments presented appear to address distinct biological processes—replication initiation versus fork reversal—rather than opposing conclusions.

Response: We agree with the reviewer that it is important to clarify that these two prominent DDK replication stress studies investigated different aspect of fork reversal and restart and also looked in different cellular context, especially BRCA-status. We have therefore clarified and toned down the according section.

Changes in manuscript: "Consistent with a role in resection of reversed forks, it was also shown that chemical inhibition of DDK, or use of CDC7 analogue sensitive (as) mutants led to a reduction of resection at reversed forks, in particular in BRCA mutant cell lines (Rainey et al., 2020). In this study, DDK is shown to act on the MRE11 nuclease, given an epistatic relationship between CDC7 inhibition and MRE11 silencing and the loss of an HU-dependent MRE11 phospho-shift on gels upon DDK inhibition (Rainey et al., 2020). Furthermore, a second study using CDC7-analog sensitive, BRCA-proficient cell lines suggested a role of DDK in stabilizing and restarting stalled replication fork structures (Jones et al., 2021). In this study, no defect in fork resection was found upon DDK inhibition, a result that can be explained by the differential BRCA status in the investigated cell lines (Jones et al., 2021; Rainey et al., 2020). Finally, additional DNA repair/replication proteins were found to be targeted by DDK using phosphoproteomics experiments, including MERIT40 (a subunit of the BRCA1-A deubiquitinase complex) and PDS5B (an accessory subunit of cohesin) (Jones et al., 2021)."

6) The question of whether DDK is essential for replication and DNA replication is controversial and central to understanding the effects of DDK inhibitors. It should be discussed with attention. In this reviewer's opinion DDK is essential for replication in human cells, however cells display a wide range of tolerance to reduced levels of origin activation. Few points below:

a) CDC7 is classified as a core essential gene in human cells (DepMap.org), and loss of CDC7 impairs proliferation in all tested cell types (>1000).
b) The potency of different DDKi greatly vary in in vitro kinase assays and proportionally in cell-based assay. When used at appropriate concentrations, all tested cell lines are sensitive to specific DDK inhibitors (including XL413).
c) DDK inhibition blocks DNA replication in the Xenopus cell-free extract system (Poh et al., Open Biol. 2013; doi:10.1098/rsob.130138). The reference currently cited as "Prokhorova et al." should be carefully checked.

Response: We thank the reviewer for the criticism and sharing their point-of-view. We agree that there is overwhelming data that DDK is critical for replication initiation in human cells. Whether it is truly essential in non-cancerous cells is less sure. The Suski et al. paper makes a compelling case (using analog-sensitive mutations, auxin-inducible degrons and gene knock-outs) that DDK is not strictly essential in non-cancerous cells, as there appear to be compensatory action by CDK1/2 (as also highlighted by reviewer #1). We are not aware of any data that would contradict this view and differences to the Xenopus cell-free extract system could be explained by evolutionary distance and/or technical differences between the experimental systems. We have modified the text to highlight the essential role of DDK in other systems and that DDK is highly important for replication initiation, but that according to the Suski et al. data proliferation of non-transformed cells is still possible without DDK (we use the term "not strictly essential"). Nonetheless, a key question remains whether a therapeutic window can be found where DDK is sufficiently active to promote proliferation of non-transformed, but not of cancer cells. We have also rewritten the final paragraph accordingly (see point 9) and hope that the reviewer agrees that we provide a balanced discussion of the topic.

Changes in manuscript: "Of note, while both DDK and CDK are essential for DNA replication in yeast and Xenopus replication systems (Hardy et al., 1997; Poh et al., 2014; Sheu and Stillman, 2010), a recent study suggests that DDK is not strictly required for proliferation in non-transformed human and mouse cells (Suski et al., 2022). The authors used different systems (analog-sensitive kinase

alleles, auxin-inducible degrons as well as knock-outs) to interfere with DDK activity/stability and observed proliferation in different non-cancerous human cell lines and mice tissues despite a strong decrease or even absence of DDK activity, suggesting that DDK can be dispensable for proliferation. Notably, the same study found Cyclin-B CDK1, known to be mainly active in late S-phase/mitosis to act redundantly with DDK for the initiation of DNA replication, and lack of both CDK1 and DDK was found to be lethal (Suski et al., 2022). This finding suggests functional redundancy of the two cell cycle kinases and that – despite not strictly essential – DDK is an important driver of proliferation. Notably, DDK was found to be required for DNA replication and cell proliferation in different cancerous mammalian cells and tumours (Iwai et al., 2019; Montagnoli et al., 2008, 2004; Rodriguez-Acebes et al., 2010). This suggests that cancer cells can become addicted to DDK. It is possible that the phenotypic differences observed in cancerous or non-cancerous cells may rely on the replication initiation function of DDK, additional functions in response to replication stress (see "DDK and replication stress") or on a combination of both."

7) No CDC7- PROTAC has yet been disclosed and the compound cited (Ji et al., 2024) is a CDK7 degrader rather than a CDC7 degrader.

Response: We thank the reviewer for pointing out this error. We have removed this statement and corrected the text accordingly.

8) The final section ("Perspectives") would benefit from substantially greater focus and clarity.

Response: We have substantially revised the Perspectives section to emphasize key conceptual advances and highlight open questions in DDK biology. For this we returned to the main approach of this review, providing the 3 perspectives of DDK function and how they are important to form a conceptual framework of DDK biology.

Changes in manuscript: "Our understanding of DDK has evolved from viewing it as a dedicated replication initiation kinase to recognizing it as a central regulator of chromosome metabolism. Understanding this broader role requires considering DDK from multiple perspectives. With this review, we make a case that DDK should be deemed a genome maintenance kinase that acts at specific locations across chromosomes and with high temporal specificity from S to G2/M phase of the cell cycle. This model raises several important conceptual questions.

First, how is DDK substrate specificity controlled? Structural studies of DDK bound to the Mcm2-7 helicase have revealed detailed docking interactions that enable highly selective phosphorylation. Whether similar docking mechanisms operate for the many additional substrates remains largely unknown. Understanding how DDK selects substrates on chromatin will be essential for dissecting its different cellular functions.

Second, how does DDK activity and or substrate selection change in response to DNA damage and replication stress? Several studies suggest that checkpoint kinases alter DDK regulation under these conditions, potentially redirecting the kinase toward new substrates involved in genome maintenance. Determining whether such substrate switching occurs and how it is controlled will be important for understanding DDK's role as a genome integrity regulator.

Third, to what extent are DDK functions conserved across organisms? While many aspects of helicase activation appear evolutionarily conserved, other functions—such as specific DNA repair roles—may

differ between yeast and vertebrates. Comparative studies across model systems will therefore remain valuable for identifying core versus organism specific mechanisms.

These questions mark promising starting points for basic biology research, at the same time they provide the intellectual fundament for DDK-targeted therapies. Here, promising preclinical data with DDK inhibitors need to be followed up with further advanced compound designs to increase the chances in clinical trials, which in the future can hopefully leverage DDK inhibition to provide new avenues in cancer therapy.”

Point-by-point response:

We thank the reviewers for their careful and thoughtful evaluation of our manuscript. We are encouraged that all reviewers now view our manuscript as a valuable and impactful resource for the field. We would like to thank them for a very productive review process that has greatly helped us to strengthen our manuscript.

Reviewers' comments:

Reviewer #1 (Remarks to the Author):

The revised manuscript presents a comprehensive and well-structured review of the functions of DDK, expanding its role from a canonical replication initiation kinase to a broader regulator of genome integrity. The main claims are clearly articulated and timely, and the synthesis of recent literature will be of strong interest to researchers working in DNA replication, replication stress, and DNA repair.

The revised version has significantly improved in clarity, balance, and conceptual framing. In particular, the authors now better contextualize the role of DDK in genome integrity pathways.

The section on Rif1-PP1 provides a clearer and more integrated view of the antagonistic relationship with DDK, and its implications beyond replication initiation are now well developed. Additionally, I like the inclusion of a dedicated perspectives section, as it outlines key open questions and helps frame the field in a more integrative manner.

Overall, the manuscript is convincing as a review article. In its current form, I believe the manuscript will be a valuable resource for the community and has the potential to influence how DDK is conceptualized within the broader context of genome maintenance.

Response: We thank the reviewer for their constructive criticism that has helped us to improve our manuscript in its revised form.

Reviewer #2 (Remarks to the Author):

Thank you for addressing the comments. This comprehensive review will serve the DDK community as well as the greater field of researchers studying cell biology.

Response: We thank the reviewer for their constructive criticism that has helped us shape this manuscript.

Reviewer #3 (Remarks to the Author):

I co-reviewed this manuscript with one of the reviewers who provided the listed reports. This is part of the Communications Biology initiative to facilitate training in peer review and to provide appropriate recognition for Early Career Researchers who co-review manuscripts.

Response: We thank the reviewer for their work in co-reviewing our paper.

Reviewer #4 (Remarks to the Author):

The authors have addressed all the comments in a constructive manner, and the revised manuscript is substantially improved in clarity, precision and overall flow. I support its publication.

I have one minor point for consideration:

Line 605: The term "creative" drug design is ambiguous, and its relevance to improving DDKi therapy is therefore unclear. I would recommend clarifying/expanding on what is required for obtaining an improvement or revising the sentence.

Response: We thank the reviewer for their constructive criticism that greatly helped us to improve the manuscript. Also, with regard to the minor point raised, we agree with the reviewer that "creative" drug design is an ambiguous formulation, which we now replaced.

Changes in manuscript text: "Therefore, alternative drug designs could become important for therapeutical DDK-inhibition."
